# A Fe Single Atom Seed‐Mediated Strategy Toward Fe_3_C/Fe—N—C Catalysts with Outstanding Bifunctional ORR/OER Activities

**DOI:** 10.1002/advs.202301656

**Published:** 2023-05-31

**Authors:** Jiangwei Chang, Qi Zhang, Jingkun Yu, Wen Jing, Siyang Wang, Guangchao Yin, Geoffrey I. N. Waterhouse, Siyu Lu

**Affiliations:** ^1^ Green Catalysis Center and College of Chemistry Zhengzhou University Zhengzhou 450001 China; ^2^ School of Physics and Optoelectronic Engineering Shandong University of Technology Zibo 255000 China; ^3^ School of Chemical Sciences The University of Auckland Auckland 1142 New Zealand

**Keywords:** carbon dots, Fe_3_C oxygen reduction reaction (ORR), Fe‐N‐C single atom catalysts, Fe_3_C, oxygen evolution reaction (OER)

## Abstract

The discovery of low‐cost and high‐performance bifunctional oxygen electrocatalysts is vital to the future commercialization of rechargeable zinc‐air batteries (ZABs). Herein, a Fe single atom seed‐mediated strategy is reported for the fabrication of Fe_3_C species closely surrounded by Fe—N_4_—C active sites with strong electronic interactions built between them and more importantly, creating optimized coordination environment, via subtly adjusting their ratio, for favorable adsorption energies of oxygen intermediates formed during oxygen reduction reaction (ORR) and oxygen evolution reaction (OER). Concretely, the voltage difference (Δ*E*) between the ORR half‐wave and OER potential at a current density of 10 mA cm^−2^ for the compositionally‐optimized Fe—N—C/Fe_3_C‐op electrocatalyst is only 0.668 V, endowing itself one of the best bifunctional OER/ORR benchmarks. As a demo, ZABs assembled with Fe—N—C/Fe_3_C‐op as the air cathode deliver a remarkable specific capacity (818.1 mAh g_Zn_^−1^) and a power density (1013.9 mWh g_Zn_^−1^), along with excellent long‐term durability (>450 h). This work extends the methodology to modulate the activity of Fe—N_4_—C atomic site, undoubtedly inspiring wide explorations on the precise design of bifunctional oxygen electrocatalysts.

## Graphical abstract

A Fe single atom seed‐mediated strategy is reported for constructing Fe—N—C/Fe_3_C composites with optimized coordination environment (Fe—N—C/Fe_3_C‐op), through subtle adjusting their ratio, for favorable adsorption‐desorption pathways of O‐containing key intermediates, thus exhibiting impressive performance with an activity descriptor ∆*E* of 0.668 V, endowing itself one of the best bifunctional oxygen evolution reaction/oxygen reduction reaction benchmarks.

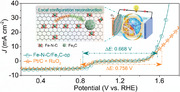

## 1 Introduction

High‐efficiency energy storage devices are vital in the transition away from polluting fossil fuel energy.^[^ ^1^, ^2^ ^]^ Among current energy storage devices under development, rechargeable zinc‐air batteries (ZABs) are highly promising due to their low cost, inherent safety, high theoretical energy density/capacity, etc.^[^ ^3^, ^4^, ^5^ ^]^ ZABs utilize the oxygen reduction reaction (ORR) at the air cathode during discharging and oxygen evolution reaction (OER) during charging process, and the kinetics of the ORR and OER determine the overall efficiency of ZABs.^[^ ^6^, ^7^ ^]^ The ORR in alkaline media occurs via a 4e^−^ pathway involving the four elementary steps and the stepwise formation of O_2_^*^, OOH^*^, O^*^, and OH^*^ intermediates (where ^*^ represents a catalytic site). OER undergoes a backward route of ORR and involves the same intermediates. As such, the misalignment about the binding energies of these O‐containing intermediates always controls the overall ORR and OER kinetics, with the discovery of high‐performance bifunctional ORR/OER electrocatalysts being challenging due to this requirement.^[^ ^7^ ^]^ Consider, for example, that Pt/C is generally regarded as the benchmark ORR electrocatalyst, while its OER performance is largely limited. IrO_2_ and RuO_2_ are the benchmark OER electrocatalysts, but they suffer from poor ORR activity. For the commercialization of rechargeable ZABs, alternative non‐precious metal electrocatalysts must be discovered, with bifunctional ORR/OER electrocatalysts capable of operating in alkaline media being especially desirable.

Recently, single‐atom catalysts (SACs), comprising isolated metal atoms incorporated into Nitrogen‐doped carbon supports have emerged as promising electrocatalysts for ORR and OER. SACs possess the advantage of near‐maximum atom‐utilization efficiency (≈100%). In particular, SACs with Fe—N—C active sites have attracted considerable attention in recent years owing to their exceptional ORR activity and further potential application in both primary and secondary ZABs.^[^ ^8^, ^9^, ^10^, ^11^, ^12^ ^]^ Whilst Fe—N—C SACs offer high intrinsic ORR activity, their bifunctional ORR/OER electrocatalytic activity remains modest. Owing to the “porphyrin‐like” Fe coordination structure, O_2_^*^ and OOH^*^ species readily adsorb on Fe—N—C site via end‐on adsorption model, with O‐O dissociation and OH^−^ desorption then proceeding, resulting in fast ORR kinetics.^[^ ^13^, ^14^ ^]^ However, conventional Fe—N—C sites bind certain oxygen intermediates too strongly or too weakly during OER, with the activity following a linear scaling relationship that inevitably leads to a minimum theoretical overpotential of ca. 0.370 V for OER.^[^ ^15^, ^16^ ^]^ The overall activity of Fe—N—C SACs can be enhanced either by improving the density of the active site or enhancing the turnover frequency of each Fe—N—C site.^[^ ^13^, ^14^ ^]^ Synthesizing Fe—N—C SACs with a high concentration of single atom site is challenging due to the competitive formation of metal nanoparticles that commonly occurs in the traditional pyrolysis used to prepare SACs.^[^ ^17^ ^]^ Accordingly, researchers are now seeking to manipulate the local geometric or electronic structures of Fe—N—C site to optimize the binding energies of ORR and OER intermediates, thereby boosting turnover frequencies.^[^ ^5^, ^7^, ^18^, ^19^, ^20^ ^]^

The adsorption/desorption behaviors of ORR/OER intermediates on a single metal site strongly depend on the interactions between the d orbital of the metal atom and the adsorbed oxygen intermediates.^[^ ^9^, ^21^, ^22^ ^]^ Accordingly, the design of bifunctional ORR/OER electrocatalysts relies on fine‐tuning the local electronic environment of Fe—N—C site. This can be achieved by introducing some synergistic metal‐based components such as metal carbides, sulfides, nitrides, nanoclusters, and nanoparticle oxides adjacent to the Fe—N—C site, which can alter its charge density and thus lower the energy barriers for ORR and OER.^[^ ^7^, ^9^, ^18^, ^23^ ^]^ Among these additives, integrating Fe—N—C with metal carbides (such as Fe_3_C) is of particular interest due to the excellent oxidation resistance and high electrical conductivity. More importantly, the high surface polarity of Fe_3_C, which results in a strong adsorption capacity for O‐containing intermediates,^[^ ^18^, ^24^ ^]^ making it particularly attractive for utilization in oxygen electrocatalysis. Fe_3_C with few‐layer graphene coating has been also shown to effectively inhibit corrosion or poisoning of Fe sites during the charging/discharging processes,^[^ ^25^ ^]^ thereby enabling long‐term operation. Surprisingly, there have been no reports to date exploring the potential of Fe—N—C/Fe_3_C composite systems as bifunctional oxygen electrocatalysts. Based on these considerations, we hypothesize that creating closed adjacent Fe—N—C and Fe_3_C heterostructures should possess unique electronic interactions that can synergistically boost the activity of Fe—N—C moiety for ORR/OER electrocatalysis. It is also worth expecting that such strong interactions should be optimized by adjusting the ratio between Fe—N—C and Fe_3_C components, thereby allowing the construction of rechargeable ZABs with excellent performance, which has not been widely explored either.

**Figure 1:**
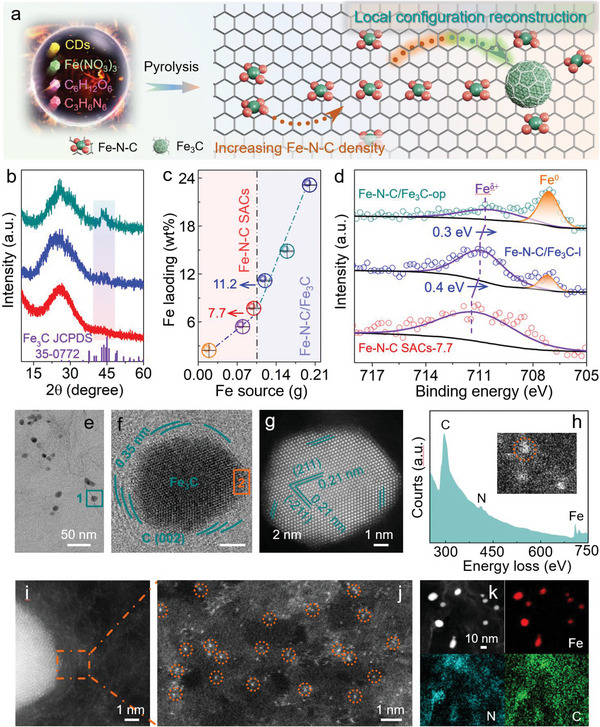
Preparation of Fe—N—C/Fe_3_C‐op electrocatalyst. a) Schematic illustration of the Fe single atom seed‐mediated route for synthesizing Fe—N—C/Fe_3_C composites. b) XRD patterns of the as‐prepared electrocatalysts, confirming that no Fe_3_C was detected until the Fe loading was > 7.7 wt%. From bottom to top: Fe—N—C SACs‐7.7, Fe—N—C/Fe_3_C‐l, and Fe—N—C/Fe_3_C‐op. c) Quantification of the Fe loading estimated by ICP‐OES analysis, implying a slow Fe increase from Fe—N—C SACs‐2.4 to Fe—N—C SACs‐7.7 with atomically dispersed Fe species, followed by a clear distinction once Fe_3_C species form. d) Deconvolution of the high‐resolution Fe 2p_3/2_ XPS spectra for Fe—N—C SACs‐7.7, Fe—N—C/Fe_3_C‐l, and Fe—N—C/Fe_3_C‐op. e) Annular bright‐field STEM image of Fe—N—C/Fe_3_C‐op. f) Magnification of area 1 in (e). g) Aberration‐corrected HAADF‐STEM image of Fe_3_C in (f). h) STEM‐EELS point spectrum of local single Fe site, marked with orange circle in the inset. i,j) Area 2 in (f) acquired by HAADF‐STEM mode, exhibiting the Fe single atoms near Fe_3_C particle. k) EDS elemental mappings of Fe—N—C/Fe_3_C‐op revealing the distribution of Fe, N, and C atoms.

In this work, we developed a Fe single atom seed‐mediated approach to synthesize Fe—N—C/Fe_3_C composite with a controlled Fe—N—C/Fe_3_C ratio that functioned as powerful bifunctional ORR/OER electrocatalysts for ZABs. Typically, the Fe—N_4_—C single atom seeds were first implanted into the N‐doped carbon skeleton until a saturated Fe loading of 7.7 wt.% was achieved. The addition of extra Fe atoms then led to the formation of Fe_3_C particles around the Fe—N_4_—C seeds during the pyrolysis for the synthesis of Fe—N—C/Fe_3_C composite (**Figure** **1**a). By this approach, the ratio of Fe_3_C to Fe—N_4_—C in the composite could be feasibly adjusted and optimized just by simply changing the amount of Fe used in the synthesis. Taking the compositionally optimized electrocatalyst (Fe—N—C/Fe_3_C‐op) as a representative example, atomically dispersed Fe coordinating with four N atoms into satellite Fe—N_4_—C sites as a local shell distributed around the Fe_3_C particle, as revealed by high‐angle annular dark‐field scanning transmission electron microscopy, ^57^Fe Mössbauer spectroscopy, X‐ray photoelectron spectroscopy (XPS), and Fe K‐edge X‐ray absorption spectroscopy (XAS). Owing to the intimate and abundant Fe—N—C/Fe_3_C interfaces, Fe—N—C/Fe_3_C‐op exhibited excellent bifunctional ORR/OER activity with fast kinetics and especially a low voltage difference (∆*E*) of 0.668 V between ORR and OER processes, outperforming almost all bifunctional ORR/OER electrocatalysts reported in the literature in alkaline media (**Table** **1**). Detailed experimental and computational studies reveal that Fe—N_4_—C and Fe_3_C can synergistically activate the adsorption of O_2_ and promote the desorption of OH^*^ intermediate, thus lowering the reaction barriers of rate‐limiting steps during ORR and OER. Finally, when Fe—N—C/Fe_3_C‐op was employed as the air cathode in a rechargeable zinc‐air battery, an excellent power density of 1013.9 mWh g_Zn_^−1^ and an outstanding peak power density of 137.4 mW cm^−2^ were obtained, with the electrocatalyst also showing long‐term durability. The seed‐mediated strategy introduced herein thus not only holds great promise for the future development of bifunctional ORR/OER electrocatalysis and high‐performance ZABs, but also sheds light on the explorations of highly active low‐cost non‐precious electrocatalysts.

**Table 1:** Performance comparison of various bifunctional ORR/OER electrocatalysts

Catalyst	ORR		∆*E* [V]	OER	Ref.
	*E*_1/2_ [V]^a)^	*n*		*E*_j10_ [V]^a)^	
Fe—N—C/Fe_3_C‐op	0.911 ± 0.004^b)^	3.85	0.668 ± 0.012^b)^	1.579 ± 0.015^b)^	This work
VNCNT arrays	N/A	N/A	0.770	N/A	[26]
Co_3_O_4_/rmGO	0.790	3.90	0.830	1.620	[27]
CNT/graphene hybrid	0.760	≈4.0	0.750	1.510	[28]
NCNTFs	0.870	3.97	0.730	1.600	[29]
SC CoO	0.850	N/A	0.710	1.560	[30]
MnCo_2_O_4_	0.850	3.94	0.830	1.630	[31]
Nd_1.5_Ba_1.5_CoFeM_n_O_9−*δ*_	0.698	3.90	0.891	1.589	[32]
PdMo bimetallene	0.950	3.95	0.750	1.700	[33]
(Co,Fe)_3_N	0.810	N/A	0.810	1.620	[34]
MoS_2_@Fe—N—C NSs	0.840	3.82	0.860	1.700	[35]
Fe,Mn/N‐C	0.928	≈4.0	0.692	1.620	[36]
S‐CFZ	0.850	≈4.0	0.680	N/A	[37]
CoNC SAC	0.860	3.70	0.790	1.650	[38]
FeMn‐DSAC	0.922	≈4.0	0.713	1.635	[39]

Note that all the listed potentials are converted to the RHE scale.

^a)^ Measured in 0.1 M KOH electrolyte

^b)^ Average data based on five independent experiments. N/A means that the information was not given in the related literature.

## 2 Results and Discussion

### 2.1 Synthesis and Structural Characterization of Fe—N—C/Fe_3_C‐op

Figure 1a depicts the single‐atom seed‐mediated strategy used to prepare the Fe—N—C/Fe_3_C composite, involving the pyrolysis of mixtures of carbon dots (CDs), glucose, iron (III) nitrate, and melamine. The Fe single atoms were pre‐incorporated into the carbon framework as seeds. In brief, CDs with an average size of ≈6 nm were first synthesized via a one‐step hydrothermal synthesis^[^ ^40^ ^]^ (Figures S1–S3, Supporting Information). The Fe—N—C SACs were then constructed based on a cascade anchoring strategy,^[^ ^14^ ^]^ wherein the as‐prepared CDs were ultrasonically dispersed in a solution containing glucose and iron(III) nitrate. In this process, the glucose molecules chelated the Fe^3+^ ions and then adsorbed on the CDs via interactions with surface functional groups. Subsequently, the dispersion was freeze‐dried to obtain solid powders, which were then ground with melamine. Pyrolysis of the resulting powder mixture yielded Fe—N—C SACs, with the synthesis involving a melamine‐derived graphitic carbon nitride intermediate^[^ ^41^ ^]^ (Figure S4, Supporting Information). Figure 1b and Figure S5, Supporting Information, show the powder X‐ray diffraction (XRD) patterns of all the Fe—N—C SACs‐x electrocatalysts (where x denotes the Fe content). All the samples exhibit two broad peaks at ca. 23° and 44°, assigned to (002) and (100) peaks of graphite, respectively. Scanning electron microscopy (SEM) and transmission electron microscopy (TEM) images show that the Fe—N—C SACs‐7.7 sample consists of micron‐sized 2D nanosheets with uniform dispersions of C, N, and Fe elements over the whole architectures without any Fe agglomerates (Figures S6–S8, Supporting Information).

By this approach, the Fe content in Fe—N—C SACs‐x electrocatalysts can be increased up to 7.7 wt.% without any obvious Fe nanoparticle formation, as determined by the combination of XRD, TEM, and inductively coupled plasma optical emission spectroscopy (ICP‐OES, Figure 1c). When the amount of Fe source is further increased, additional diffraction peaks attributable to a Fe_3_C phase emerge (Figure 1b). The electrocatalysts prepared at these higher Fe loadings, containing both Fe—N—C and Fe_3_C, are denoted herein as Fe—N—C/Fe_3_C‐l, Fe—N—C/Fe_3_C‐op, and Fe—N—C/Fe_3_C‐h (where the subscripts l, op, and h denote a low, optimum and high ratio of Fe_3_C to Fe—N—C site, with these designations based on ^57^Fe Mössbauer measurements). Results indicate that Fe atoms first coordinate with N‐doping sites on the carbon support during the pyrolysis, and the remaining Fe atoms form Fe_3_C species once these N sites are filled, as confirmed by TEM images (Figures S9 and S10, Supporting Information). Figure 1c and Table S1, Supporting Information, show how the Fe loading, which is determined by ICP‐OES, changes as a function of the amount of Fe source. The Fe loading in the samples increases slowly with Fe source on going from Fe—N—C SACs‐2.4 to Fe—N—C SACs‐7.7, followed by a steep increase on going from Fe—N—C/Fe_3_C‐l to Fe—N—C/Fe_3_C‐h (the latter being associated with Fe_3_C phase formation), revealing their intrinsically different growth behaviors. This result is consistent with the deconvolution of the high‐resolution Fe 2p XPS. As shown in Figure 1d and Figures S11 and S12, Supporting Information, only oxidized Fe specie in the form of Fe^*δ*+^ is detected until Fe—N—C SACs‐7.7, accompanied by the arising of Fe 2p_3/2_ orbitals of zero‐valence Fe (originating from metallic Fe_3_C) from Fe—N—C/Fe_3_C‐l to Fe—N—C/Fe_3_C‐h. Meanwhile, the Fe^*δ*+^ peak for Fe—N—C/Fe_3_C‐l presents an upshift by 0.40 eV compared with that in Fe—N—C SACs‐7.7, possibly indicating the possible electronic interactions between Fe—N—C and Fe_3_C, which is also evidenced by the N 1s XPS spectra (see more details in Figure S13, Supporting Information). Next, aberration‐corrected high‐angle annular dark‐filed scanning TEM (HAADF‐STEM) was applied to further investigate the distribution of Fe species at the atomic‐scale resolution. As displayed in Figure 1e–g and Figure S14, Supporting Information, several Fe_3_C particles fairly anchor on carbon support, and the lattice spacing of 0.21 nm in two directions also matches well with the (211) crystalline plane of Fe_3_C phase.^[^ ^24^ ^]^ Furthermore, it is noteworthy that Fe_3_C are mainly encapsulated by few‐layer graphene (Figure S15, Supporting Information), which can prevent the Fe atoms from corrosion during the ORR/OER process. More importantly, Figure 1h clearly exhibits that abundant isolated Fe atoms with the colocation of Fe and N, evidenced by the STEM‐electron energy loss spectroscopy (EELS), closely distribute around Fe_3_C (Figure 1i,j), suggesting the coexistence of Fe_3_C and satellite Fe single atoms. EDX elemental mappings also confirm the homogenous distribution of Fe, N, and C atoms in Fe—N—C/Fe_3_C‐op (Figure 1k). The presence of Fe_3_C and Fe single atom sites in close proximity, separated by few‐layer graphene, provides a perfect interface for electronic “cross‐talk” and modification of the Fe—N—C electronic structure, as can be seen by XPS. It should be noted that the introduction of the Fe_3_C species did not increase the defect concentration in the carbon support (as discussed in detail in Figure S16, Supporting Information), further revealing the Fe single atom seed‐mediated local construction of Fe_3_C species. Furthermore, the high specific surface area and mesoporosity should be helpful to mass and charge transfer during the ORR/OER process, leading to the outstanding electrocatalytic activity of Fe—N—C/Fe_3_C‐op (Figure S17, Supporting Information).

### 2.2 Atomic‐Scale Structure Analysis of Fe—N—C/Fe_3_C‐op by X‐Ray Absorption Spectroscopy (XAS) and ^57^Fe Mössbauer Spectroscopy

**Figure 2:**
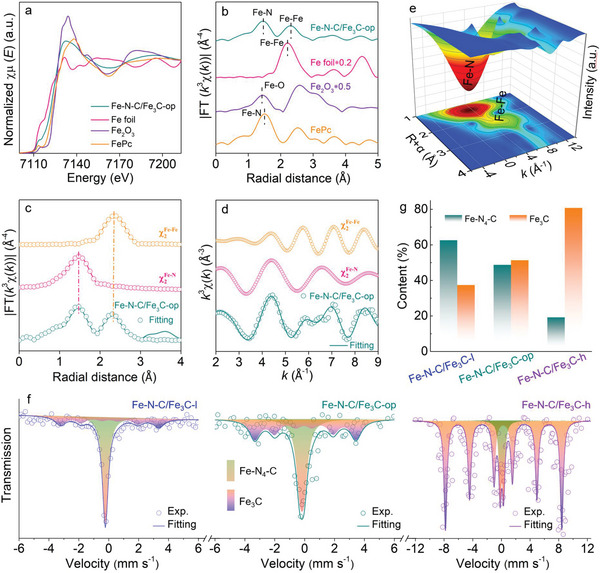
Atomic structure analyses of Fe—N—C/Fe_3_C‐op electrocatalyst. a) Fe K‐edge XANES spectra, where commercial Fe foil, Fe_2_O_3_, and FePc were applied as the reference samples. b) Normalized Fe K‐edge FT‐EXAFS spectra for Fe—N—C/Fe_3_C‐op and selected reference samples. c,d) Fe K‐edge EXAFS fitting curves for Fe—N—C/Fe_3_C‐op in (c) R and (d) k space. e) k^3^‐weighted Fe K‐edge WT‐EXAFS contour plot for Fe—N—C/Fe_3_C‐op. f) Room‐temperature ^57^Fe Mössbauer spectra of Fe—N—C/Fe_3_C‐op, where the data of Fe—N—C/Fe_3_C‐l and Fe—N—C/Fe_3_C‐h are also included for comparison. Exp.: experimental data. g) Bulk quantitative analysis of the Fe—N_4_—C and Fe_3_C contents in Fe—N—C/Fe_3_C‐l, Fe—N—C/Fe_3_C‐op, and Fe—N—C/Fe_3_C‐h based on ^57^Fe Mössbauer data.

Following confirmation of Fe_3_C and Fe single atoms in the Fe—N—C/Fe_3_C‐op electrocatalyst by HAADF‐STEM characterization, XAS experiments including Fe K‐edge X‐ray absorption near‐edge structure (XANES) and extended X‐ray absorption fine structure (EXAFS) analyses were carried out to probe the valency and local coordination of Fe. As shown in the Fe K‐edge XANES spectra (**Figure** **2**a), the pre‐edge curve of Fe—N—C/Fe_3_C‐op is very similar to that of iron (II) phthalocyanine (FePc), which has well‐defined Fe—N_4_ coordinated site. This means that Fe state in Fe—N—C/Fe_3_C‐op SAC should be similar to that in FePc. Besides, the pre‐edge curve of Fe—N—C/Fe_3_C‐op is located between that of Fe foil and Fe_2_O_3_ reference samples, further indicating that the Fe valence state in Fe—N—C/Fe_3_C‐op was likely +2. Figure 2b shows the corresponding *k* ^3^‐weighted Fourier transform EXAFS (FT‐EXAFS) spectra. Compared with Fe foil and FePc, the Fe—N—C/Fe_3_C‐op shows a primary peak at ≈2.30 Å together with a shoulder peak at around 1.45 Å, corresponding to the Fe—Fe and Fe—N scattering paths,^[^ ^5^, ^14^, ^42^ ^]^ respectively. These results confirm the coexistence of zero‐valent Fe in Fe_3_C and Fe single atoms in a Fe—N—C coordination for Fe—N—C/Fe_3_C‐op. The local Fe coordination was further explored by fitting the least‐squares EXAFS curves to obtain the structural parameters. As shown in Figure 2c,d, and Figures S18 and S19, Supporting Information, the fittings match well with the experimental data, with the isolated Fe atoms in Fe—N—C/Fe_3_C‐op possessing an average coordination number by N of 4.2 and an average Fe—N bond distance of 1.94 Å (see more details in Table S2, Supporting Information). Taken together, these findings confirm the presence of Fe—N_4_—C moieties in Fe—N—C/Fe_3_C‐op. More powerful Fe K‐edge wavelet transform‐EXAFS (WT‐EXAFS) was also employed to investigate the Fe environment in Fe—N—C/Fe_3_C‐op and selected reference samples due to its high resolution in both *k* and *R* spaces (Figure 2e, Figure S20, Supporting Information). Specifically, the wavelet transform maximum in the WT‐EXAFS contour plot of Fe—N—C/Fe_3_C‐op can be confidently assigned to the Fe—N and Fe—Fe bonding, with these WT signals being similar to those observed for FePc and Fe foil, respectively. The data further confirms that Fe—N—C/Fe_3_C‐op possesses Fe—N—C/Fe_3_C interfaces, wherein atomically dispersed Fe in Fe—N_4_—C sites is in close proximity to neighboring Fe_3_C particles and therefore likely activated.

Room‐temperature ^57^Fe Mössbauer spectroscopy was also used to identify and determine the content of various kinds of Fe species in the different electrocatalysts. As exhibited in Figure 2f, all the curves of Fe—N—C/Fe_3_C‐l, Fe—N—C/Fe_3_C‐op, and Fe—N—C/Fe_3_C‐h can be fitted with two components, corresponding to Fe—N_4_—C site and Fe_3_C species,^[^ ^5^, ^14^, ^43^, ^44^ ^]^ respectively. Quantitative analyses revealed that Fe—N—C/Fe_3_C‐l contained 62.6% Fe—N_4_—C and 37.4% Fe_3_C by absorption area, Fe—N—C/Fe_3_C‐op contained 48.7% Fe—N_4_—C and 51.3% Fe_3_C, and finally, Fe—N—C/Fe_3_C‐h contained 29.2% Fe—N_4_—C and 70.8% Fe_3_C (fitting parameters are listed in Table S3, Supporting Information). The data confirms that the content of Fe_3_C in the catalysts progressively increased with the Fe loading in the catalysts. The increase in Fe_3_C content thus altered the charge of the Fe^*δ*+^ cations in the Fe—N_4_—C sites, as can be seen in the high‐resolution Fe 2p_3/2_ spectra (Figure 1d). Considering that Mössbauer technique can characterize the bulk information of materials, the Fe single atom seed‐mediated synthetic strategy used in this work allows the creation of Fe—N—C/Fe_3_C interfaces with tunable compositions, providing a robust platform for precisely regulating the electron state of Fe—N_4_—C site towards optimized ORR/OER bifunctional activity (as verified by experiment below).

### 2.3 Electrocatalytic Activity Evaluation

**Figure 3:**
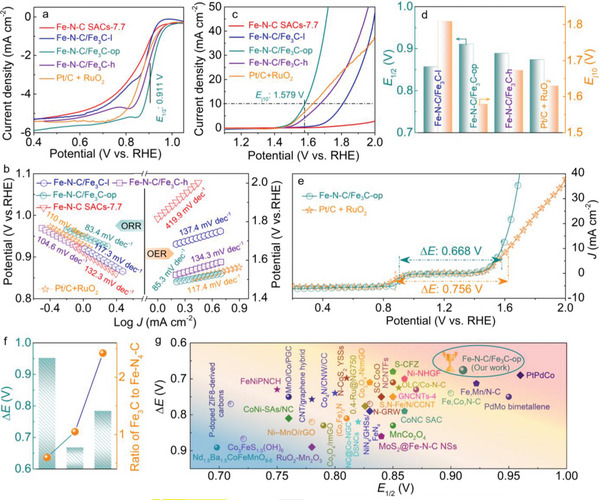
Bifunctional activity evaluation. a) ORR LSV curves. b) Corresponding Tafel plots for ORR and OER on Fe—N—C SAC‐7.7, Fe—N—C/Fe_3_C‐l, Fe—N—C/Fe_3_C‐op, Fe—N—C/Fe_3_C‐h, and Pt/C + RuO_2_ electrocatalysts. c) OER LSV curves. All potentials were calibrated to the RHE. d) *E*_1/2_ and *E*_j10_ of these measured electrocatalysts. e) Bifunctional LSV curves tested in a 0.10 m KOH solution, showing the superior activity of Fe—N—C/Fe_3_C‐op towards both ORR and OER relative to Pt/C + RuO_2_. f) Correlation between the value of ∆*E* and Fe_3_C/Fe—N_4_—C ratio, revealing the optimal ratio in Fe—N—C/Fe_3_C‐op. g) Comparison of the bifunctional ORR/OER activity of Fe—N—C/Fe_3_C‐op and other recently reported electrocatalysts (Table S5, Supporting Information), in which the x and y axes represent *E*_1/2_ and ∆*E*, respectively.

Rechargeable ZABs require air‐electrode electrocatalysts to offer excellent ORR and OER activity and stability.^[^ ^45^, ^46^, ^47^ ^]^ To evaluate the performance of the prepared electrocatalysts, electrocatalytic ORR and OER tests were carried out in a three‐electrode system applying O_2_‐saturated 0.1 m KOH electrolyte, where the benchmarked Pt/C + RuO_2_ electrocatalysts were served as the reference. As shown in the linear sweep voltammetry (LSV) curves using the rotating ring‐disk electrode (RRDE) technique (Figure S21, Supporting Information), the ORR activity is progressively enhanced from Fe—N—C SACs‐2.4 to Fe—N—C SACs‐7.7 and the half‐wave potential (*E*_1/2_) is improved to 0.837 V versus reversible hydrogen electrode (RHE), indicating that the ORR activity could be boosted by increasing the loading of Fe—N_4_—C active site, in good accord with reported literature.^[^ ^13^, ^14^ ^]^ The ORR performance was further improved after introducing Fe_3_C species, with Fe—N—C/Fe_3_C‐l exhibiting a more positive *E*_1/2_ of 0.857 V versus RHE (**Figure** **3**a), comparable to that of the Pt/C + RuO_2_ benchmark with the *E*_1/2_ of 0.874 V versus RHE. Note that by regulating the electronic structure of Fe—N_4_—C by increasing the Fe_3_C to Fe—N_4_—C ratio, the ORR activity can be further improved with Fe—N—C/Fe_3_C‐op exhibiting an outstanding *E*_1/2_ of 0.911 V versus RHE, surpassing that of the Pt/C + RuO_2_ electrocatalyst. However, the value of *E*_1/2_ decreases to 0.889 V versus RHE for Fe—N—C/Fe_3_C‐h with the continuously increased Fe_3_C/ Fe—N_4_—C ratio (Table S4, Supporting Information). These results show an optimal amount of Fe_3_C is highly beneficial for tuning the electronic structure of Fe—N_4_—C sites, leading to enhanced ORR activity. Tafel plots were additionally obtained based on the LSV curves to evaluate the kinetics of ORR. As presented in Figure 3b, Fe—N—C/Fe_3_C‐op offers the smallest Tafel slope of 83.4 mV dec^−1^, signifying it owns the fastest ORR kinetics and best ORR activity among all the tested electrocatalysts. To obtain deeper insights into the ORR process, RRDE tests and the Koutecky–Levich (K–L) method were applied to study ORR on Fe—N—C/Fe_3_C‐op at different rotation speeds (from 400 to 1600 rpm). The obtained K–L plots were linear and parallel (Figure S22, Supporting Information), suggesting a first‐order reaction.^[^ ^7^ ^]^ According to the K–L equation, the number of transferred electrons (*n*) on Fe—N—C/Fe_3_C‐op during ORR was calculated to be 3.85 with a very low peroxide (H_2_O_2_) yield of less than 5.0% over a wide range of ORR potentials (Figure S23, Supporting Information), indicating a dominant four‐electron ORR pathway and excellent selectivity for electroreduction of O_2_ to OH^−^. Furthermore, Fe—N—C/Fe_3_C‐op exhibits superior tolerance to methanol poisoning and excellent stability without significant changes in electronic environment for the active Fe sites (Figures S24–S27, Supporting Information) compared to the Pt/C + RuO_2_ electrocatalyst. Synergistic effects between the Fe—N_4_—C and Fe_3_C species were determined critical to the excellent all‐round ORR performance of Fe—N—C/Fe_3_C‐op (see details in Figure S28, Supporting Information).

In stark sharp, these Fe—N—C SACs‐x electrocatalysts containing only atomically dispersed Fe—N_4_—C sites exhibited poor OER activity (Figure S29, Supporting Information), consistent with the work reported by other researchers.^[^ ^7^, ^48^, ^49^ ^]^ Regulation of the local coordination environment of metal centers in SACs can significantly enhance the OER performance.^[^ ^18^, ^21^, ^50^, ^51^, ^52^, ^53^ ^]^ As expected, the introduction of foreign Fe_3_C species leads to a remarkable improvement of the OER activity (Figure 3c), evidenced by the relatively low potential (*E*_j10_) of 1.809 V versus RHE required to achieve a current density of 10 mA cm^−2^ on Fe—N—C/Fe_3_C‐l. For Fe—N—C/Fe_3_C‐op, *E*_j10_ value is only 1.579 V versus RHE, comparable to that of Pt/C + RuO_2_ (1.63 V). Interestingly, a similar *E*_j10_‐related volcano‐type variation trend to that of *E*_1/2_ has also been confirmed for Fe—N—C/Fe_3_C‐l, Fe—N—C/Fe_3_C‐op, and Fe—N—C/Fe_3_C‐h (Figure 3d), meanwhile indicative of the optimized Fe_3_C/Fe—N—C ratio towards OER activity. Consistently, Fe—N—C/Fe_3_C‐op also affords the lowest Tafel slope of 85.3 mV dec^−1^ (Figure 3b), the smallest charge transfer resistance, and the highest electrochemical surface area of 21.8 mF cm^−2^ (Figure S30, Supporting Information) in comparison with other electrocatalysts and even lower than that of Pt/C + RuO_2_ (117.4 mV dec^−1^), revealing its faster OER kinetics.

The bifunctional ORR/OER performance of electrocatalysts can be quantified by the ∆*E* descriptor,^[^ ^6^, ^7^ ^]^ which is widely defined as the potential difference between *E*_j10_ and *E*_1/2_. As shown in Figure 3e, Fe—N—C/Fe_3_C‐op delivers a low ∆*E* of only 0.668 V, outperforming Pt/C + RuO_2_ (∆*E* = 0.756 V) and the other electrocatalysts tested in this work (Figures S31 and S32, Table S4, Supporting Information). Also, the rational ratio of Fe_3_C to Fe—N—C in Fe—N—C/Fe_3_C‐op is regarded as the key to its optimal bifunctional activity (Figure 3f). Despite this, what attracts us more is that the performance of Fe—N—C/Fe_3_C‐op outperforms most of the reported bifunctional electrocatalysts (Figure 3g, Table S5, Supporting Information), whose ∆*E* are generally higher than 0.70 V. Clearly, the constructed Fe—N—C/Fe_3_C‐op serves well as a highly promising precious metal‐free electrocatalyst, instead of Pt/C and RuO_2_, to fulfill excellent ORR/OER bifunctional performance, validating the Fe single atom seed‐mediated strategy reported herein towards fabricating improved SACs‐based electrocatalysts for rechargeable ZABs.

### 2.4 Insights into the Underlying ORR/OER Mechanism

**Figure 4:**
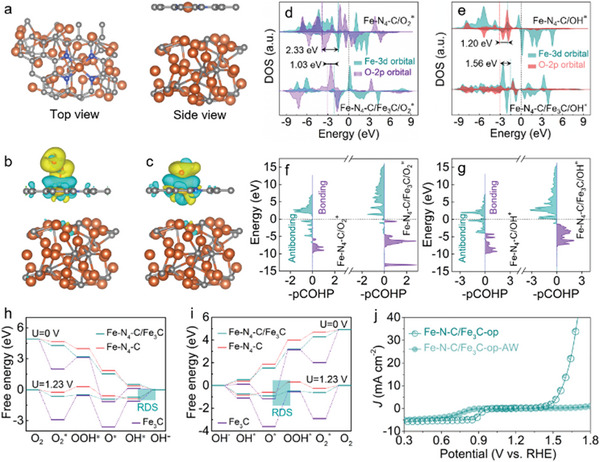
Theoretical and experimental studies unveiling the mechanism of enhanced bifunctional ORR/OER activity. a) Structural model of Fe_3_C‐modified Fe—N_4_—C electrocatalyst (Fe—N_4_—C/Fe_3_C) built for theoretical calculation. b) Electron density difference plots for the Fe—N_4_—C/Fe_3_C model with adsorbed O_2_ (O_2_^*^) and c) adsorbed OH^−^ (OH^*^). Cyan and yellow contours signify charge depletion and accumulation in real space, respectively. d,e) Calculated DOSs for Fe—N_4_—C and Fe—N_4_—C/Fe_3_C with (d) O_2_^*^ and (e) OH^*^, in which the Fermi level is indicated by the dashed black line. f) Calculated pCOHP diagrams for O_2_^*^ and g) OH^*^, confirming the critical promoting role of Fe_3_C in O_2_ adsorption and OH^*^ desorption on Fe—N_4_—C/Fe_3_C. h,i) ∆*G* diagrams for (h) ORR and (i) OER on Fe—N_4_—C, Fe—N_4_—C/Fe_3_C, and Fe_3_C at U = 0 and 1.23 V, respectively. j) Comparison of LSV curves between Fe—N—C/Fe_3_C‐op and Fe—N—C/Fe_3_C‐op‐AW, demonstrating that the synergistic effect between Fe_3_C and Fe—N—C components is responsible for enhanced bifunctional ORR/OER activity.

To further uncover the roles of Fe—N_4_—C and Fe_3_C species in the bifunctional ORR/OER activity of Fe—N—C/Fe_3_C‐op, first‐principles‐based density functional theory (DFT) calculations on models of Fe—N_4_—C, Fe_3_C exposing a (211) lattice plane and Fe—N_4_—C with adjacent Fe_3_C (Fe—N_4_—C /Fe_3_C) after geometry optimization (**Figure** **4**a, Figures S33 and S34, Supporting Information) were performed. It has been well considered that the transition metal centers in SACs typically have a high binding energy for the O_2_ molecule and a high energy barrier for four‐electron ORR/OER processes, resulting in poor bifunctional activity.^[^ ^7^, ^13^, ^42^ ^]^ Based on the electrocatalytic tests, the interfacial effect induced by the introduction of Fe_3_C was expected to lower the energy barriers for ORR and OER on Fe—N_4_—C sites. As shown in Figure S35, Supporting Information, electron‐rich Fe_3_C acts as the donor providing 0.06 e^−^ to electron‐withdrawing Fe—N_4_—C site. The electron donation from Fe_3_C likely modulates the adsorption‐desorption of O‐related intermediates on the Fe—N_4_—C sites, thus enabling reversible ORR/OER to proceed efficiently. To verify this hypothesis, we calculated the electron density difference for Fe—N_4_—C/Fe_3_C with adsorbed O_2_* and OH* (where * denotes the active site), considering these are the key intermediates for the ORR and OER processes. Clearly, O_2_ adsorption on Fe—N_4_—C site is effectively promoted (−0.708 eV for Fe—N_4_—C/Fe_3_C vs −0.314 eV for Fe—N_4_—C model) after introducing Fe_3_C (Figure S36, Table S6, Supporting Information), with the O—O bond length for O_2_ on Fe—N_4_—C/Fe_3_C (1.285 Å) being elongated compared to that on Fe—N_4_—C (1.265 Å) and O_2_ molecule (1.234 Å). Results suggest that Fe—N_4_—C/Fe_3_C is more effective in activating the O_2_ molecule (Table S7, Supporting Information). Electron density difference plots show that charge transfer from Fe_3_C to Fe—N_4_—C enhanced electronic interactions between Fe—N_4_—C and O_2_* and OH* (Figure 4b,c, Figure S37, Supporting Information), facilitating the generation of the OOH* intermediate. To further qualitatively explore the electronic effects in the bond strength, the density of states (DOSs) about O_2_* and OH* on the Fe—N_4_—C/Fe_3_C and Fe—N_4_—C models were calculated. As shown in Figure 4d, the overlap between the Fe 3d and O 2p orbitals of O_2_* (1.03 eV) on Fe—N_4_—C/Fe_3_C is smaller than that on the Fe—N_4_—C model (2.33 eV), validating stronger adsorption of O_2_ on Fe—N_4_—C/Fe_3_C. In contrast, a larger overlap is observed for OH* on Fe—N_4_—C/Fe_3_C (1.56 eV) compared to OH* on Fe—N_4_—C (1.20 eV, Figure 4e), suggesting that Fe—N_4_—C/Fe_3_C has a lower energy barrier for OH* desorption. This has been further evidenced in the projected crystal orbital Hamilton population (pCOHP) profile and corresponding integrated COHP (ICOHP), where the O_2_*—Fe bond value decreased from −0.75 to −1.79 on going from Fe—N_4_—C and Fe—N_4_—C/Fe_3_C, whilst the value for the OH*—Fe bond is increased from −2.96 to −2.34, respectively (Figure 4f,g, Table S8, Supporting Information). Results demonstrate the promoting role of Fe_3_C species in strengthening O_2_ adsorption and facilitating OH* desorption in Fe—N_4_—C/Fe_3_C.

Figure 4h shows the Gibbs free energy (∆*G*) diagram of the elementary steps in the four‐electron ORR process for the different model catalysts (Figures S38–S40, Supporting Information). A consistent downhill energy pathway is observed on both the Fe—N_4_—C and Fe—N_4_—C/Fe_3_C models at 0 V, indicating a spontaneously exothermal process, while Fe_3_C model has energy barriers of 1.12 (O_2_^*^ + H_2_O + e^−^ → OOH^*^ + OH^−^) and 1.27 eV (O^*^ + H_2_O + e^−^ → OH^*^ + OH^−^). When the potential is increased to 1.23 V, the highest uphill ∆*G* for the Fe—N_4_—C/Fe_3_C and Fe—N_4_—C models is the fourth reaction step (OH^*^ + e^−^ → OH^−^), representing the rate‐determining step (RDS) in the ORR process. For the Fe_3_C model, the RDS was the O^*^ to OH^*^ reaction. The limiting energy barriers for each model are as follows: Fe—N_4_—C/Fe_3_C (0.73 eV), Fe—N_4_—C (0.86 eV), and Fe_3_C (2.50 eV). Results imply that the introduction of Fe_3_C enhances the kinetics of ORR on Fe—N_4_—C site. Furthermore, DFT calculations of ∆*G* pathways for the OER show that the RDS is the third step (O^*^ + OH^−^ + e^−^ → OOH^*^) for all models (Figure 4i) with Fe—N_4_—C/Fe_3_C exhibiting a lower limiting energy barrier (0.42 eV) than Fe—N_4_—C (0.89 eV) and Fe_3_C (3.05 eV). Clearly, the addition of Fe_3_C was highly beneficial for promoting the OER activity of Fe—N_4_—C/Fe_3_C. More importantly, the key role of Fe_3_C as ORR/OER promoters has also been experimentally verified that the bifunctional reactivity can be significantly weakened after acid washing of the Fe—N—C/Fe_3_C‐op electrocatalyst for selectively removing the Fe_3_C nanoparticles (denoted herein as Fe—N—C/Fe_3_C‐op‐AW, Figure 4j and Figures S41–S43, Supporting Information). Taken together, the synergy created by interfacial charge transfer from Fe_3_C to Fe—N_4_—C site can optimize the adsorption‐desorption behaviors of key ORR/OER intermediates and lower limiting energy barriers for these processes, imparting Fe—N—C/Fe_3_C‐op with outstanding bifunctional ORR/OER activity.

### 2.5 Application in ZABs

**Figure 5:**
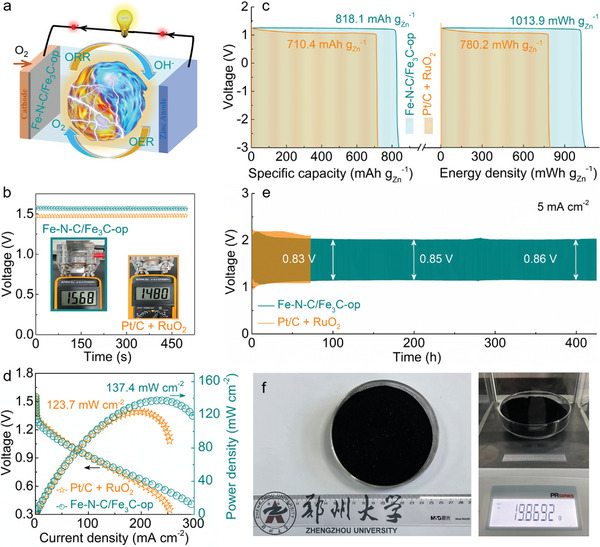
Performance of rechargeable ZABs. a) Schematic diagram of the Fe—N—C/Fe_3_C‐op‐based ZABs. b) Open‐circuit voltage test. The inset shows the *V*_OC_ of the assembled ZABs. c) Specific capacity and energy‐density curves of ZABs fabricated with Fe—N—C/Fe_3_C‐op and Pt/C + RuO_2_ electrocatalysts. d) LSV profiles and power density curves of the ZABs with Fe—N—C/Fe_3_C‐op and Pt/C + RuO_2_. e) Charge‐discharge cycling curves of rechargeable ZABs with Fe—N—C/Fe_3_C‐op and Pt/C + RuO_2_ electrocatalysts at a constant current density of 5 mA cm^−2^. f) Photograph of ≈20 g production of the Fe—N—C/Fe_3_C‐op in a batch synthesis.

Inspiringly, the superb ORR/OER bifunctional electrocatalytic activity of the as‐constructed Fe—N—C/Fe_3_C‐op promises its practical application as the air cathode in rechargeable ZABs (**Figure** **5**a) with an open‐circuit potential (*V*_OC_) of 1.568 V (Figure 5b), higher than that of Pt/C + RuO_2_‐based counterpart (1.480 V). As exhibited in Figure 5c, the ZABs using Fe—N—C/Fe_3_C‐op electrocatalysts deliver a specific capacity of 818.1 mAh g_Zn_^−1^ (close to the theoretical value of 821 mAh g_Zn_^−1^) with a corresponding energy density of 1013.9 mWh g_Zn_^−1^ (theoretical value of 1086 mWh g_Zn_^−1^), superior to that of Pt/C + RuO_2_–assembled ZABs (710.4 mAh g_Zn_^−1^ and 780.2 mWh g_Zn_^−1^, respectively). Also, LSV polarization profiles (Figure 5d) show that the ZABs with Fe—N—C/Fe_3_C‐op as air cathode electrocatalyst exhibit lower polarization during the charging/discharging process compared to the counterpart using Pt/C + RuO_2_. Correspondingly, the ZABs containing Fe—N—C/Fe_3_C‐op electrocatalyst offer an excellent peak power density of 137.4 mW cm^−2^, which is 10% higher than that of ZABs with Pt/C + RuO_2_ and outperforms most of the recently reported bifunctional ORR/OER electrocatalysts (Table S5, Supporting Information). Furthermore, the ZABs with Fe—N—C/Fe_3_C‐op exhibit long‐term stability (lifespan > 450 h) and meanwhile maintain a low voltage gap of 0.86 V (Figure 5e). In contrast, poor cycling stability of the ZABs with the Pt/C + RuO_2_ electrocatalyst is demonstrated with an unsatisfactory life span. Additionally, the synthesis of the low‐cost Fe—N—C/Fe_3_C‐op electrocatalyst could easily be scaled up to meet large‐scale demand (Figure 5f), thus holding great promise for use in commercial rechargeable ZABs applications.

In summary, a Fe single‐atom seed‐mediated strategy is proposed to fabricate Fe—N—C/Fe_3_C composites with atomically dispersed Fe—N_4_—C sites electronically modulated by closely adjacent Fe_3_C species. The intrinsic synergistic effect between Fe—N_4_—C and Fe_3_C can accelerate sluggish ORR and OER kinetics and more importantly, the optimized ratio between them has been demonstrated critical for constructing robust ORR/OER bifunctional electrocatalysts in alkaline media. As such, the obtained Fe—N—C/Fe_3_C‐op electrocatalyst exhibits excellent bifunctional performance with ∆*E* of 0.668 V, surpassing most of the analogous bifunctional electrocatalysts. Moreover, theoretical analyses reveal that, with the electron‐donating characteristic of Fe_3_C, the electron state of Fe in Fe—N_4_—C is fine‐tuned for favorable adsorption of O_2_ and desorption of OH^*^, thereby lowering the energy barriers for both ORR and OER processes. Rechargeable ZABs with Fe—N—C/Fe_3_C‐op cathode afford a high power density of 137.4 mW cm^−2^, low charging/discharging polarizations, and excellent stability over 1150 cycles (ca. 450 h). This work not only demonstrates the untapped potential of SACs as low‐cost bifunctional ORR/OER electrocatalysts for ZABs but also enlightens the precise design of targeted active sites for various energy‐related applications.

## Conflict of Interest

The authors declare no conflict of interest.

## Supporting information

Supporting Information

## Data Availability

The data that support the findings of this study are available from the corresponding author upon reasonable request.
