# Supplementary material for: A Fe Single Atom Seed‐Mediated Strategy Toward Fe3C/Fe—N—C Catalysts with Outstanding Bifunctional ORR/OER Activities
Source: Adv Sci (Weinh). 2023 May 31;10(22):2301656. doi: 10.1002/advs.202301656 (PMC10401088; doi:10.1002/advs.202301656)
Supplement: Supplementary file 1 — Supporting Information [file ADVS-10-2301656-s001.pdf]

ADVANCED SCIENCE
Open Access

Supporting Information

for *Adv. Sci.*, DOI 10.1002/advs.202301656

A Fe Single Atom Seed-Mediated Strategy Toward Fe_3C/Fe–N–C Catalysts with Outstanding Bifunctional ORR/OER Activities

Jiangwei Chang, Qi Zhang, Jingkun Yu, Wen Jing, Siyang Wang, Guangchao Yin, Geoffrey I. N. Waterhouse and Siyu Lu**

Supporting Information

A Fe single atom seed-mediated stratery towards Fe_3C/Fe-N-C catalysts with outstanding bifunctional ORR/OER activities

Jiangwei Chang[1,4], Qi Zhang[2,4], Jingkun Yu[1,4], Wen Jing[1], Siyang Wang[1], Guangchao Yin,[2,*] Geoffrey I. N. Waterhouse,[3] and Siyu Lu[1,*]

[1] Green Catalysis Center and College of Chemistry, Zhengzhou University, Zhengzhou 450001, P.R. China.

[2] School of Physics and Optoelectronic Engineering, Shandong University of Technology, Zibo, 255000, P.R. China.

[3] School of Chemical Sciences, The University of Auckland, Auckland 1142, New Zealand.

[4] These authors contributed equally: Jiangwei Chang, Qi Zhang, Jingkun Yu.

[*] Corresponding author: S.Y.L. (email: sylu2013@zzu.edu.cn)

Methods

Synthesis of carbon dots (CDs). CDs were prepared through a conventional hydrothermal method reported by our group.[1, 2] By varying the hydrothermal temperature and time, the size distribution of the CDs could be controlled. After the hydrothermal syntheses, the as-obtained CDs dispersions were centrifuged, filtered and finally freeze dried to obtain solid CDs with an average size of 6 nm, based on the statistic results by HR-TEM.

Synthesis of Fe-N-C SACs-x electrocatalysts. All the preparation of the samples were performed referring to a previously reported method[3] with slight modifications. Firstly, 0.3 g glucose monohydrate and 0.02 g iron (III) nitrate nonahydrate were dissolved in 30 mL of deionized water under continuous stirring for 30 min. Then, 20 mL of the as-prepared CDs dispersion was poured into the solution, after which the water was evaporated in a water bath at 100 ^{o}C. The obtained solid was ground with 4.0 g melamine to obtain a precursor powder, which was then heated in a tube furnace under N_2 flow (50 sccm) from room temperature to 800 ^{o}C at a heating rate of 2 ^{o}C/min. After heating at 800 ^{o}C for 2 h, the sample was allowed to naturally cool to room temperature under a N_2 flow. The product obtained is denoted herein as Fe-N-C SACs-2.4, where the 2.4 indicates the Fe content in the sample determined by ICP-OES. Fe-N-C SACs-5.5, and Fe-N-C SACs-7.7 electrocatalysts were also prepared only by increasing the mass of iron (III) nitrate nonahydrate in a same procedure.

Synthesis of Fe-N-C/Fe_3C-l, Fe-N-C/Fe_3C-op and Fe-N-C/Fe_3C-h electrocatalysts. The syntheses of the Fe-N-C/Fe_3C-l, Fe-N-C/Fe_3C-op and Fe-N-C/Fe_3C-h electrocatalysts were similar to that of Fe-N-C SACs-2.4t, except that the mass of iron (III) nitrate nonahydrate used in the syntheses were increased. Following the increased mass of Fe source, the excess Fe atoms would spontaneously gather to form Fe_3C species near the atomically dispersed Fe sites due to the limited availability of N-anchoring sites. The labels of l, op and h in Fe-N-C/Fe_3C-l, Fe-N-C/Fe_3C-op and Fe-N-C/Fe_3C-h signify a low, optimal and high ratio of Fe_3C to Fe-N-C sites (determined by ^{57}Fe Mössbauer spectroscopy), respectively, in relation to the bifunctional oxygen activity.

Synthesis of control sample (Fe-N-C/Fe_3C-op-AW). To understand synergetic effects between Fe-N-C and Fe_3C species in the Fe-N-C/Fe_3C-op electrocatalyst, the as-synthesized

Fe-N-C/Fe_3C-op was dispersed in an aqueous 1 M H_2SO_4 solution at 60 °C for 12 h to adequately remove the unstable Fe_3C species. The acid-treated product was then collected by filtration, washed with deionized water until the washing were neutral, and finally dried at 80 °C overnight to obtain the Fe-N-C/Fe_3C-op-AW electrocatalyst (in which AW denotes acid washed).

Characterization. The crystalline structures of the samples were characterized using a wide-angle power XRD diffractometer (Rigaku Ultima IV) equipped with the Cu K_α radiation. UV-vis measurements were performed on a TU-1810PC. The morphology of the as-obtained materials were obtainedstudied by field emission SEM (JEOL S4800). PL emission and corresponding PL excitation spectra of the CDs dispersion were obtained on a RF-6000 instrument. HAADF-STEM and bright field images were taken under an acceleration voltage of 80 kV by an aberration-corrected JEOL ARM200F, configurated with a Gatan EDAX energy-dispersive X-ray (EDX) system and a Gatan Enfina electron energy-loss spectrometer (EELS). XPS was conducted to probe the surface chemical information of the samples by using a Kratos AXIS Ultra DLD spectrometer equipped with an Al Kα source. The chemical composition and electronic structure of the materials were characterized by FTIR (Thermo Fisher Nicolet iN10) and Raman (HORIBA Scientific LabRAM HR). The Fe loading in all samples were acquired by ICP-OES (ICP-OES Agilent 720ES). Room-temperature ^{57}Fe Mössbauer spectra of the electrocatalysts and reference samples were recorded on a SEE Co W304 Mössbauer spectrometer, adopting a ^{57}Co/Rh source in the transmission geometry. The Mössbauer data were fitted using MossWinn 4.0 software. Fe K-edge X-ray absorption spectroscopy (XAS) measurements were performed on beamline BL14W1 at the Shanghai Synchrotron Radiation Facility (SSRF). All data were collected in transmission mode at room temperature using a Si(111) double-crystal monochromator to control the photon energy. A Fe foil was used for photon energy calibration and background subtraction/normalization were carried out with Athena software. The fitting of the EXAFS spectra used the Artemis and IFEFFIT software packages.

Electrochemical Measurements. The electrochemical activity of all electrocatalysts were evaluated in a standard three-electrode system and the signals were recorded using a CHI760E-controlled electrochemical workstation. Rotating disk electrode (RDE) and rotating ring disk electrode (RRDE) measurements used a system supplied by Pine Research Instruments,

consisting of a rotating disk electrode as the working electrode (Φ = 5 mm with an area of 0.196 cm^{-2}), a saturated calomel electrode (SCE) as the reference electrode, and a platinum wire as the counter electrode, respectively. The recorded potentials were calibrated to RHE using the following formula (1):

$$E_{RHE} = E_{SCE} + 0.241 + 0.0592\times pH \quad (1)$$

in which E_{RHE} and E_{SCE} represent the potentials relative to the RHE and SCE, respectively.

For the preparation of the working electrodes, the as-prepared electrocatalysts were coated onto the disk electrode by drop-casting. Briefly, 3 mg of the electrocatalyst, 500 μL of absolute ethanol and 50 μL of Nafion (5 wt.%) solution were mixed together and ultrasonicated in a cold water bath for 30 min to obtain an uniform catalyst ink. Then, 30 μL of the ink was pipetted onto a polished disk electrode. The samples were then allowed to dry naturally in air, after which the working electrode was used for electrochemical tests.

The ORR activity tests on all electrocatalysts were conducted at room temperature in an O_2-saturated 0.1 M KOH aqueous electrolyte. Prior to these experiments, the electrolyte was continually bubbled with O_2 for 30 min. The rotating speed of the disk electrode was maintained at 1600 rpm during the ORR measurements. The bifunctional oxygen activities of the electrocatalysts were evaluated using an iR corrected LSV curve at a scan rate of 10 mV s^{-1}. The potential during the ORR test was set from 0.177 to −1.177 V vs. SCE, whilst the potential was set between 0 and 0.8 V vs. SCE for OER measurements. The half-wave potential ($E_{1/2}$), which is defined as the potential corresponding to half of the limiting current density, was used as a measure of the ORR activity. The potential (E_{j10}) at a current density of 10 mA cm^{-2} was used as measure of the OER activity. The ORR/OER bifunctional oxygen electrocatalytic activity of the electrocatalysts were calculated as the voltage difference (ΔE) between Ej_{10} and $E_{1/2}$ (equation 2):

$$\Delta E = E_{j10} - E_{1/2} \quad (2)$$

Tafel plots were constructed to evaluate the ORR and OER kinetics on the basis of the LSV curves, with the values calculated according to Tafel equation (3):

$$\eta = a + blogj \quad (3)$$

where a represents the intercept, b is the Tafel slope, j is the current density, and η is the potential.

For the ORR test on the RDE, the working electrode was cathodically scanned at a rate of 5mV s^{-1} with the rotating speed varying from 400 to 1600 rpm in O_2-saturated 0.1 M KOH electrolyte. The electron transfer number (n) per O_2 molecule for ORR can be calculated using Koutechy-Levich (K-L) equation (3 and 4):

$$\frac{1}{J} = \frac{1}{J_K} + \frac{1}{J_L} = \frac{1}{J_K} + \frac{1}{B\omega^{1/2}} \quad (4)$$

$$B = 0.62nFC_0D_0^{2/3}v^{-1/6} \quad (5)$$

where J denotes the measured current density; J_k and J_L are the kinetic and limiting current densities, respectively; ω is the angular velocity of the disk electrode (ω = $2\pi r/60$, r is the rotational speed of the RDE); C_0 is the volumetric concentration of the O_2 molecule ($C_0 = 1.2 \times 10^{-6}$ mol cm^{-3}); F is the Faraday constant (F = 96485 C mol^{-1}); D_0 is the diffusion coefficient of O_2 ($D_0 = 1.9 \times 10^{-5}$ cm^2 s^{-1}) and v is the kinematic viscosity of the electrolyte (v = 0.01 cm^2 s^{-1}).

RRDE measurements were further performed to evaluate the four-electron selectivity during ORR. Measurements were performed at a scan rate of 10 mV s^{-1}. The rotating disk electrode potential was set as 0.392 V vs. SCE. The hydrogen peroxide (H_2O_2) yield and electron transfer number (n) were calculated based on the following equations (6 and 7):

$$H_2O_2\ (\%) = 200 \times \frac{I_R/N}{I_D + I_R/N} \quad (6)$$

$$n = 4 \times \frac{I_D}{I_D + I_R/N} \quad (7)$$

Where I_D denotes the disk current, I_R is the ring current, and N represents the ring collection efficiency. Here, the value of N was determined to be 0.37.

Rechargeable ZABs tests. Electrocatalyst inks consisting of catalyst (8 mg) and super C (1 mg) were ultrasonically dispersed in 2 mL of ethanol and 20 μL of Nafion (5 wt.%). For the ZABs tests, the as-prepared ink was uniformly coated onto the carbon cloth and then dried at 80 ^{o}C for 6 h to prepare the air cathode, achieving a mass loading of 1 mg cm^{-2}. A Zn plate was used as the anode and assembled with the air cathode into ZABs with an aqueous electrolyte consisting of 6 M KOH and 0.2 M $Zn(OAc)_2$. Polarization curves were recorded on a CHI760E electrochemical workstation and CT2001A testing system were used to evaluate the performance of the fabricated ZABs. During the galvanostatic charging-discharging process, the ZABs were charged for 10 min, held in the charged state for 1 min, and then discharged for 10 min at a current density of 5 mA cm^{-2}. According to the galvanostatic discharge curves and the consumed mass of the Zn plate, the

specific capacity and energy density of the ZABs were calculated.

Computational details. Spin-polarized density functional theory (DFT)[4, 5] calculations were performed using the Vienna Ab Initio Simulation package (VASP).[6, 7] Projector augmented wave (PAW) pseudopotentials in conjunction with the Perdew-Burke-Ernzerhof (PBE) generalized gradient approximation (GGA) were employed for the calculations.[8, 9] A plane wave basis set with a kinetic energy cutoff of 400 eV was used to expand the wave functions. Structures in our calculations were allowed to relax to a tolerance in the atomic force at 0.02 eV/Å. The Brillouin zone was sampled by a 2 × 2 × 1 Monkhorst-Pack grid.[10] The DFT-D3 empirical correction method was employed to describe the van der Waals interactions. A 15 Å vacuum layer was set on the z-axis to avoid interactions between periodic slabs. Considering the Coulomb interactions in Fe-d states, a Hubbard correction was added (U_{eff} = 3 eV for the d-states of Fe atoms).

The adsorption energies of the ORR intermediates were calculated according to the following equation:

$$\Delta E_{\mathrm{ads}} = E_{\mathrm{ads}}{}^{*} - E_{\mathrm{surface}} - E_{\mathrm{ads}}$$

where $E_{\mathrm{ads}}{}^{*}$ is the energy of the surface with adsorbed molecules, whilst E_{surface} and E_{ads} represent the energies of the surface model and isolated molecules, respectively.

Data availability

The data that support the findings of this work are available in the Supporting material. Source data are provided with this paper.

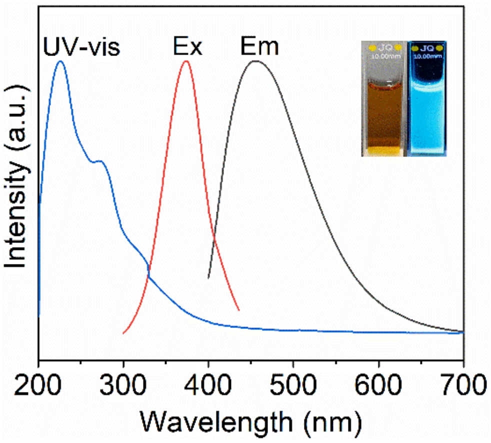

Figure S1. Excitation-dependent UV−vis absorption spectrum, photoluminescence (PL) emission spectrum and excitation spectrum of the as-prepared CDs dispersion after purification by dialysis.

When the CDs dispersion was subjected to 360 nm irradiation, the solution showed strong blue luminescence with an obvious excitation-dependent PL behaviour, confirming the successful synthesis of CDs.

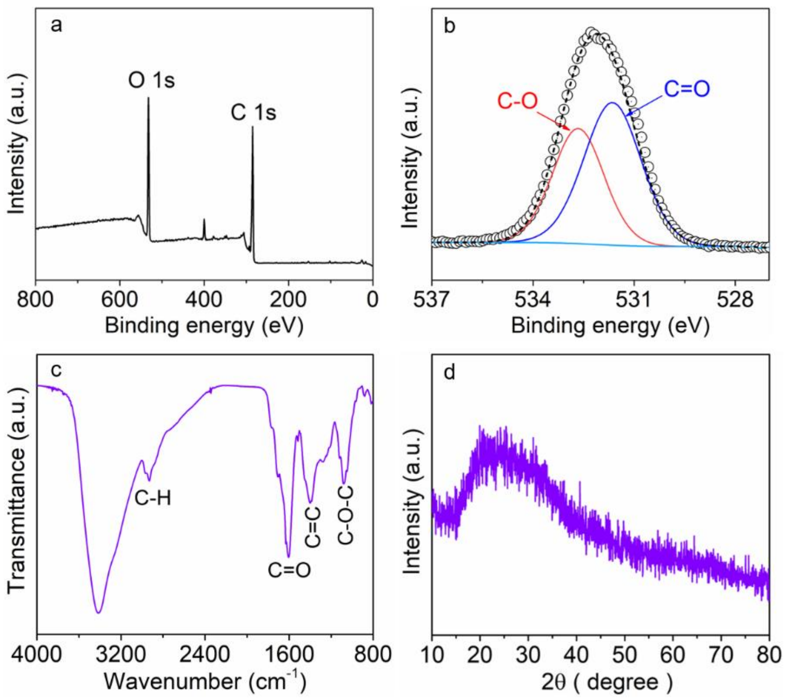

Figure S2. (a) XPS survey spectrum of CDs, showing the presence of C (26.1 at%) and O (67.1 at%). (b) Deconvolution of high-resolution O 1s spectrum of CDs, exhibiting that O-containing functional groups mainly contain O singly bound to carbon (C−O) and O doubly bound to carbon (C=O),[11, 12] respectively. (c) Fourier transform infrared spectroscopy (FT−IR) spectrum and (d) X−ray diffraction (XRD) patterns of CDs.

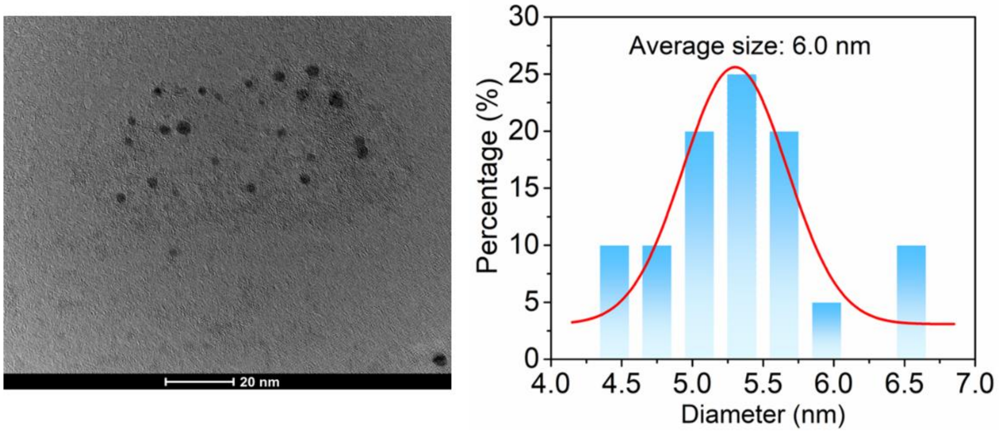

Figure S3. HRTEM images of the as-prepared CDs and corresponding particle size distribution, showing an average particle size of ca. 6.0 nm.

In the synthesis of CDs, a dialysis process was essential to remove the impurities and to achieve a uniform CDs size. Generally, decreasing the CDs size can improve the surface/volume ratio and thus offer a high concentration of superficial functional groups to efficiently anchor glucose molecules.[3, 13] As such, theoretically, the smaller the size of CDs is, the better. However, with the CDs size decreasing to sub-nanometer, the dialysis tube with a low cutoff molecule should be adopted for the purification of CDs, which inevitably leads to the co-existence of some impurities in the resultant CDs products. Moreover, CDs with larger size will decrease the number of adsorption sites for anchoring single metal atom. Based on these considerations, it was found that the optimized average size of CDs is 6 nm in this study.

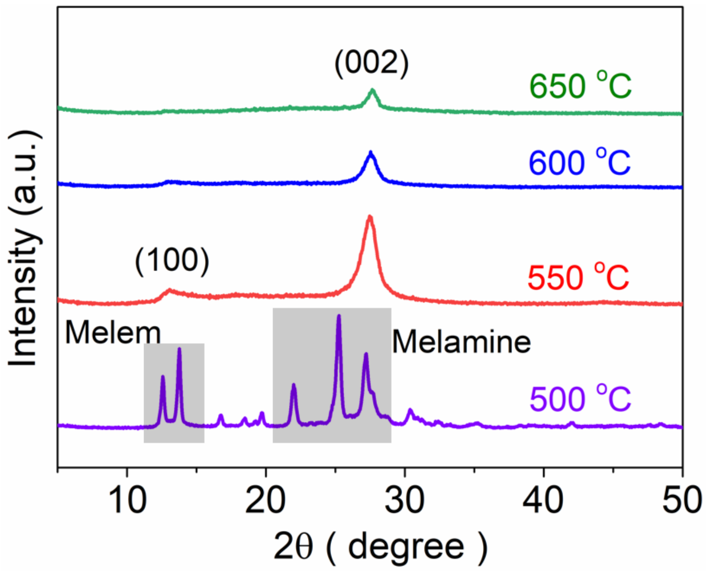

Figure S4. XRD results of the intermediates at different temperatures and the structural transformations of melamine.

During pyrolysis, melamine molecules undergo condensation to form polymers, networks and the final polymeric carbon nitride (g-C_3N_4, two typical (100) and (002) diffraction peaks stemming from interplanar packing of s-triazine unit and π–π interlayer stacking of g-C_3N_4 can be clearly observed[14]) at approximately 550 °C. Above 600 °C, these g-C_3N_4 becomes unstable and continuously dissociate, accompanied with the generation of nitrogen and cyano-containing fragments[3], to complete decomposition.

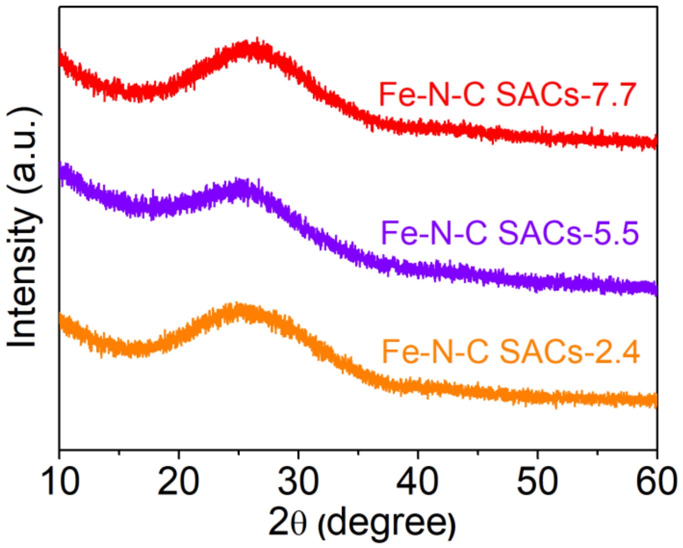

Figure S5. XRD patterns of the as-prepared Fe-N-C SACs-x electrocatalysts.

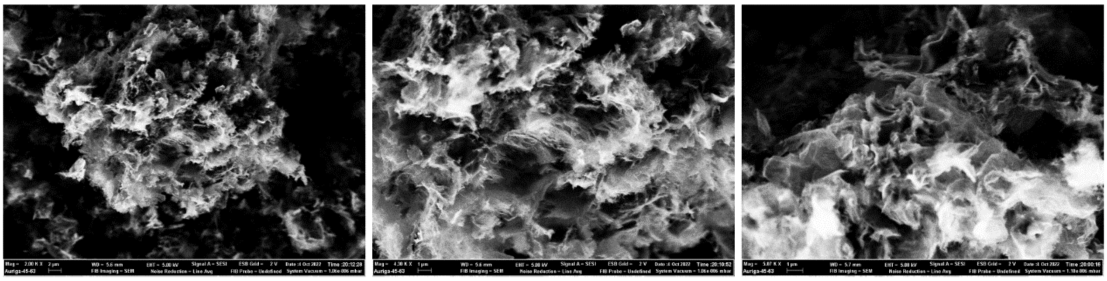

Figure S6. SEM images of Fe-N-C SACs-7.7.

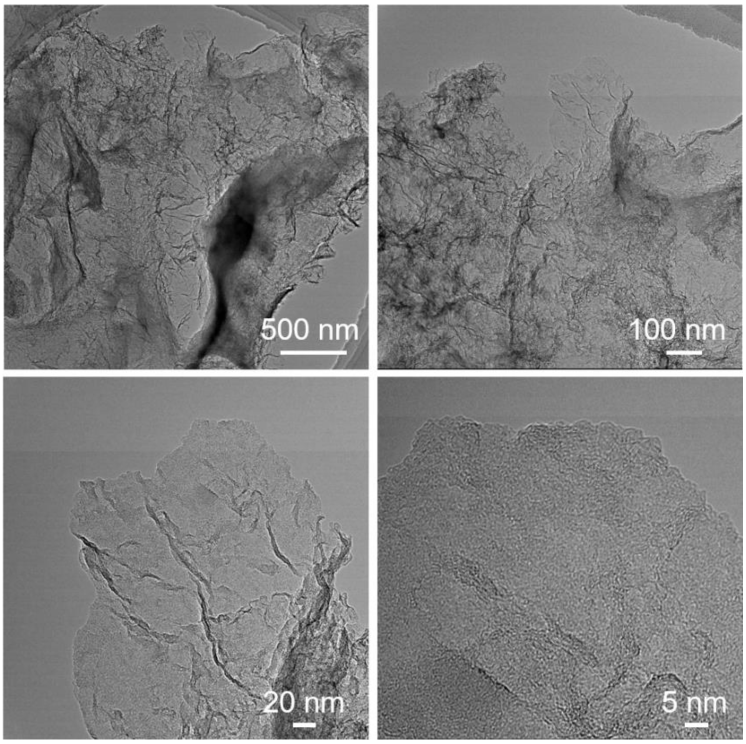

Figure S7. TEM images of Fe-N-C SACs-7.7.

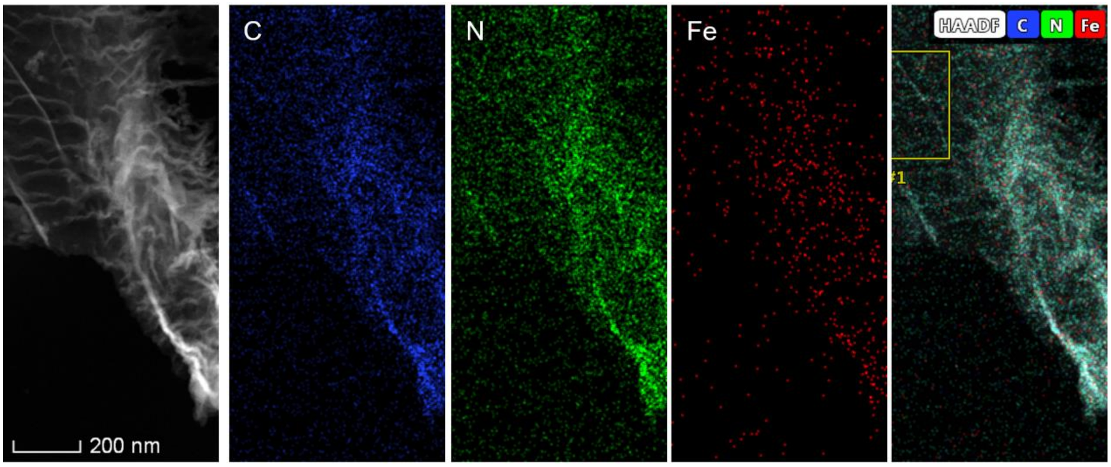

Figure S8. EDX elemental mapping images of Fe-N-C SACs-7.7.

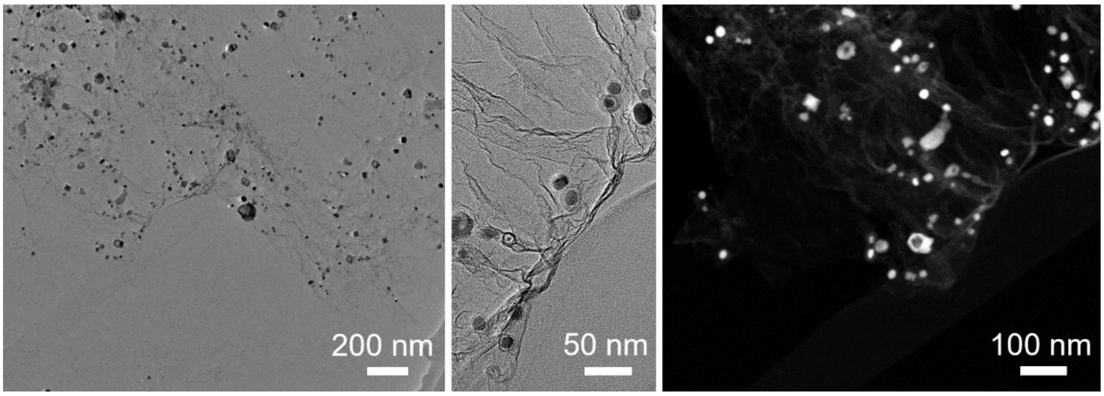

Figure S9. TEM and HAADF images of Fe-N-C/Fe_3C-l.

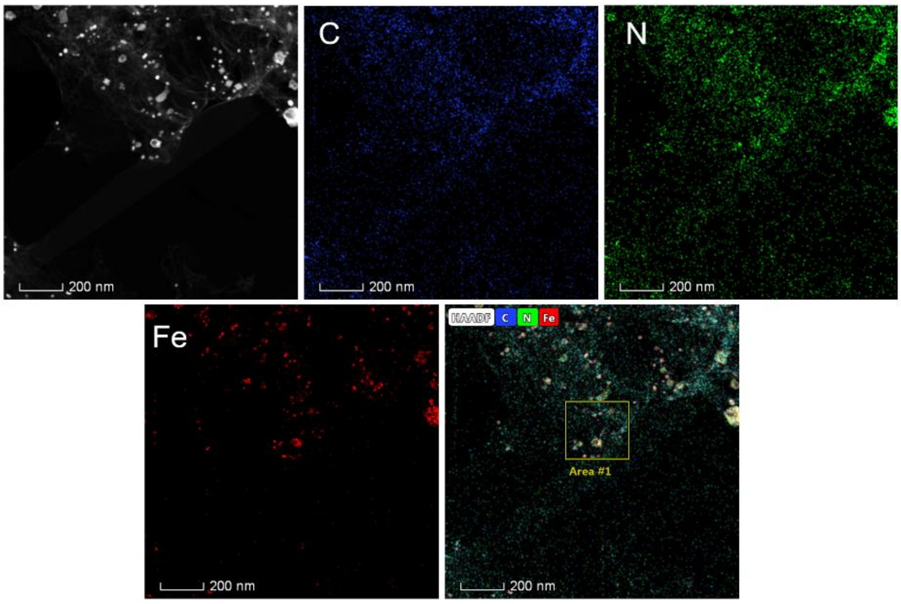

Figure S10. EDX elemental mapping images of Fe-N-C/Fe_3C-l.

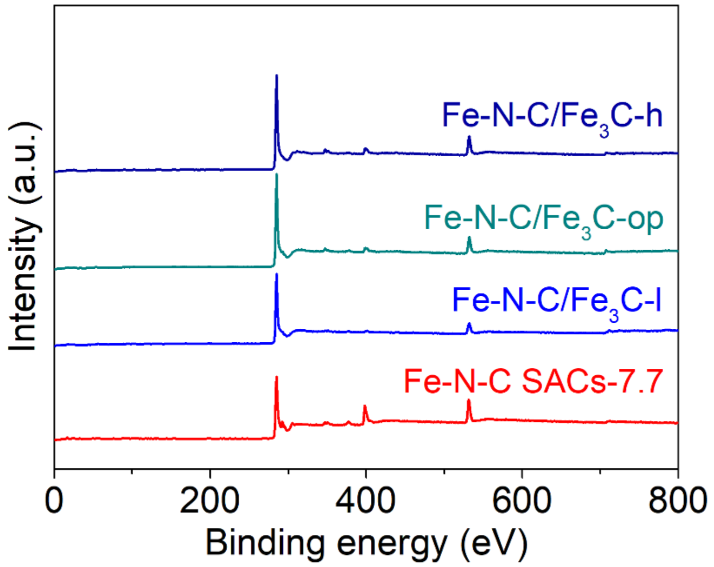

Figure S11. XPS survey spectra for the as-prepared samples.

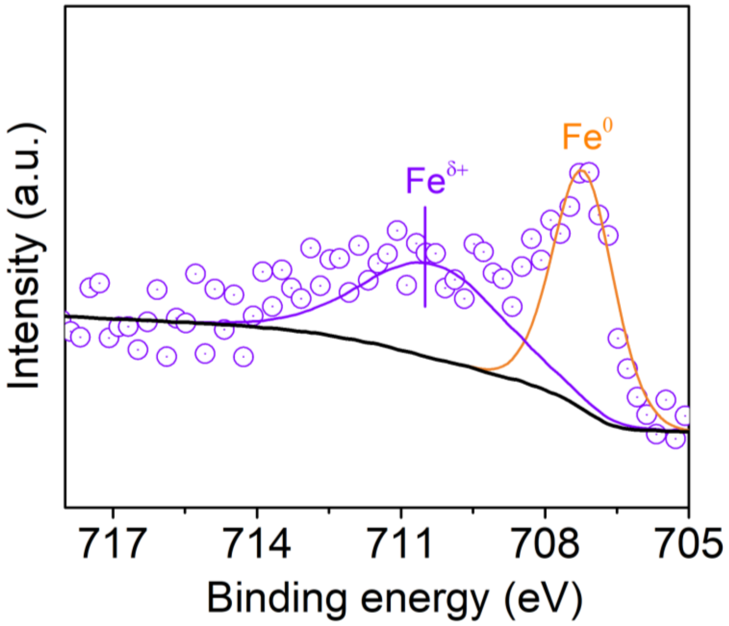

Figure S12. High-resolution Fe $2p_{3/2}$ spectrum for Fe-N-C/Fe_3C-h. The peak at ca. 707.5 eV assigned to the Fe $2p_{3/2}$ signal of a metallic Fe_3C species.

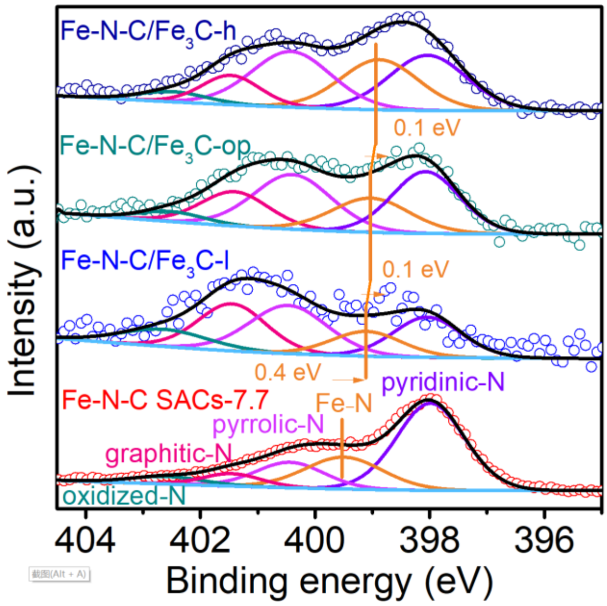

Figure S13. High-resolution N 1s XPS spectra for the as-prepared samples.

XPS measurements were applied to explore the chemical state of N elements on the surface of these electrocatalysts. Clearly, the high-resolution N 1s XPS spectra can be deconvoluted into pyridinic-N, Fe-N bonding, pyrrolic-N, graphitic-N, and oxidized-N,[15-17] respectively, implying that N atoms are doped into the carbon lattice. The relatively intense peak in Fe-N-C SACs-7.7 at 399.6 eV is attributed to the Fe species via coordination with N atoms, indicative of the possible presence of Fe single atoms in the form of Fe-N-C sites[18] in the N-doped carbon materials. Notably, this peak position gradually shifts to a lower binding energy for Fe-N-C/Fe_3C-l, Fe-N-C/Fe_3C-op and Fe-N-C/Fe_3C-h in comparison with that of Fe-N-C SACs-7.7, most likely signifying the electronic interaction between Fe-N-C and Fe_3C sites.

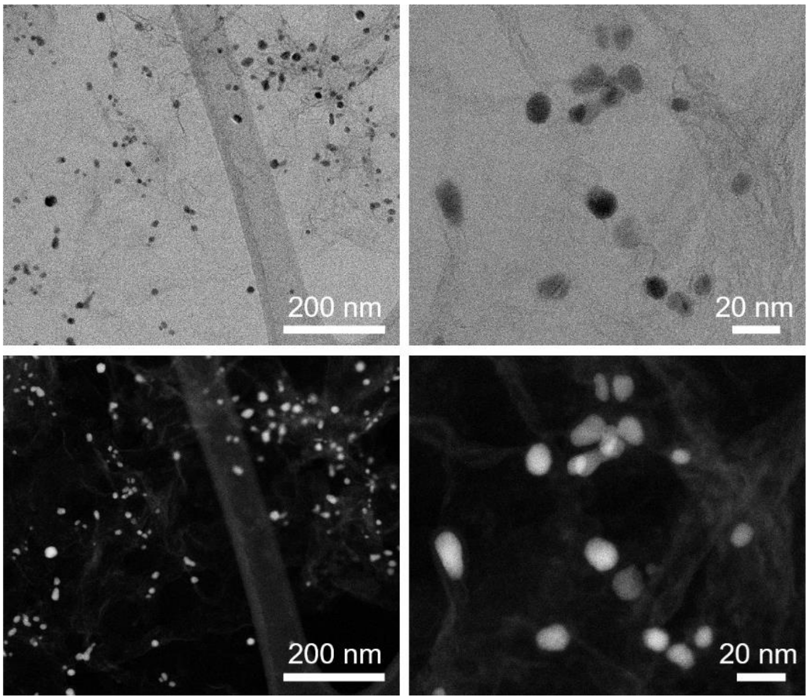

Figure S14. HAADF-STEM images of Fe-N-C/Fe_3C-op.

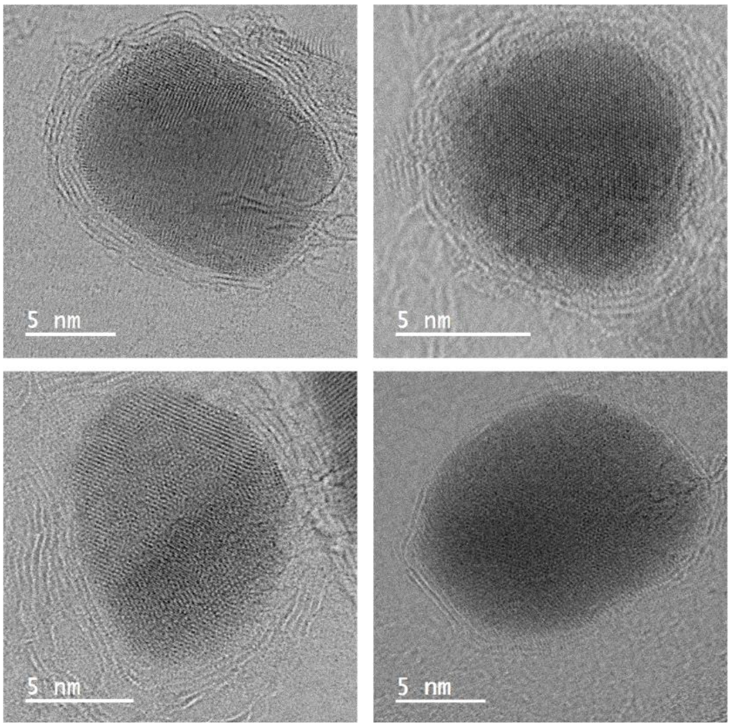

Figure S15. Representative STEM images of Fe-N-C/Fe_3C-op presenting few-layer graphene-coated Fe_3C particles.

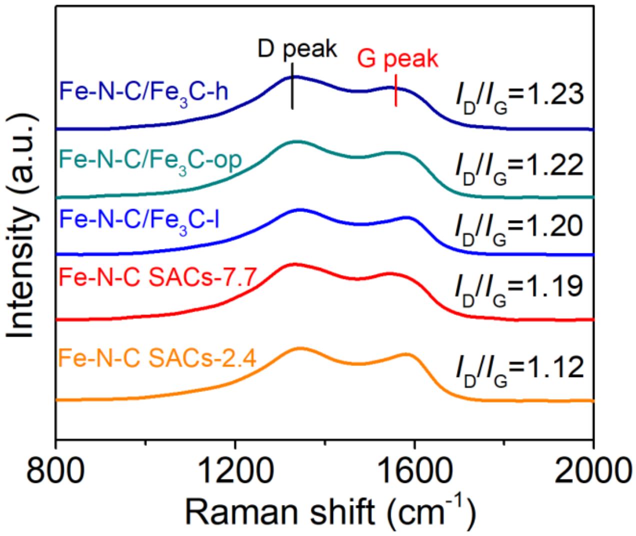

Figure S16. Raman spectra of Fe-N-C SACs-2.4, Fe-N-C SACs-7.7, Fe-N-C/Fe_3C-l, Fe-N-C/Fe_3C-op and Fe-N-C/Fe_3C-h.

The doping of foreign species into the carbon lattices generally create carbon defects, with the Raman I_D/I_G ratio frequently applied to evaluate the structural irregularity[19, 20]. For the Fe-N-C SACs-x electrocatalysts, the I_D/I_G ratio increased rapidly as the Fe loading increased, signifying increased disorder in the graphene layers (i.e. more defects in the hexagonal-C lattice). However, it is interesting to find that the value of I_D/I_G will not significatly increase from Fe-N-C SACs-7.7 to Fe-N-C/Fe_3C-SACs-l as the Fe loading increased and the ratio also increased more slowly from Fe-N-C/Fe_3C-l to Fe-N-C/Fe_3C-h electrocatalysts contained increasing amounts of Fe_3C species around the Fe-N_4-C sites. Results sugeest that the formation of Fe_3C had little impact on the carbon network, possibly suggesting that these Fe_3C mostly generate in the local regions with the pre-planted Fe-N-C sites.

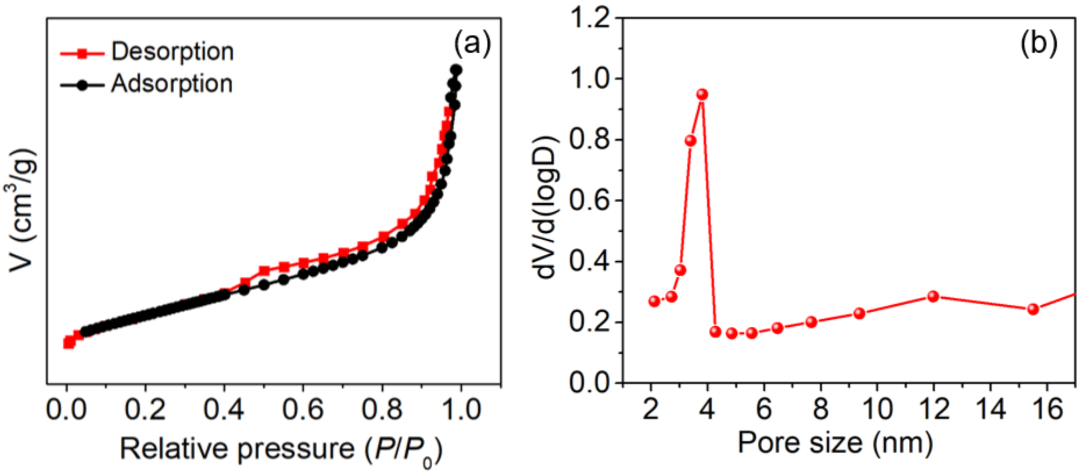

Figure S17. (a) Nitrogen sorption-desorption isotherms and (b) the pore size distributions of Fe-N-C/Fe_3C-op.

The N_2 adsorption−desorption isotherms of Fe-N-C/Fe_3C-op exhibits a type IV isotherm characteristic of mesoporous materials. The Brunauer−Emmett−Teller (BET) specific surface area of the Fe-N-C/Fe_3C-op reaches up to 338.8 m^2 g^{-1}, indicating a large number of accessible active sites. Moreover, the pore size distribution clearly exhibits the mesopore feature of the Fe-N-C/Fe_3C-op. Such high specific surface area and mesoporosity should be conducive to outstanding electrocatalytic activity.

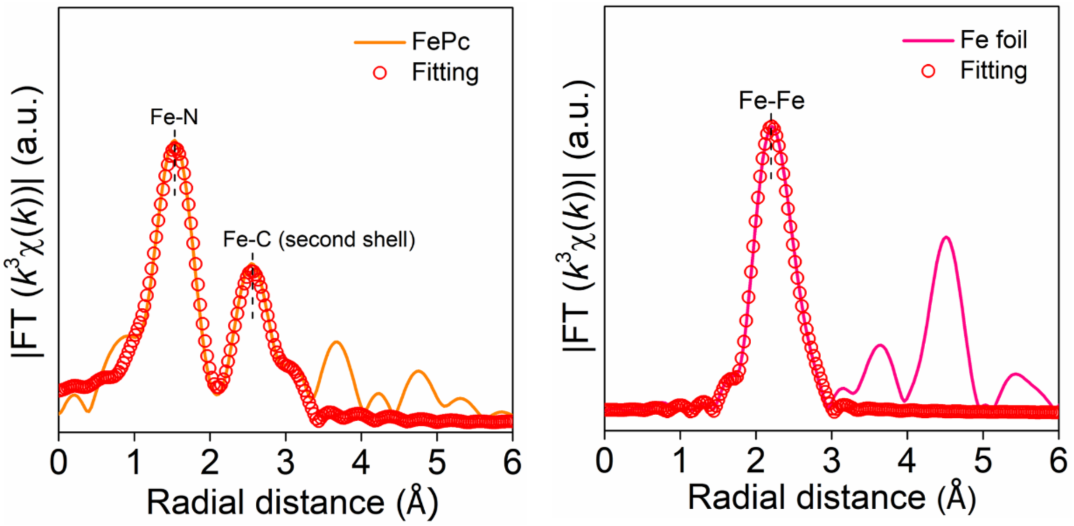

Figure S18. Fe K-edge EXAFS spectra in *R* space and the corresponding fitting curves for the FePc and Fe foil reference samples.

.

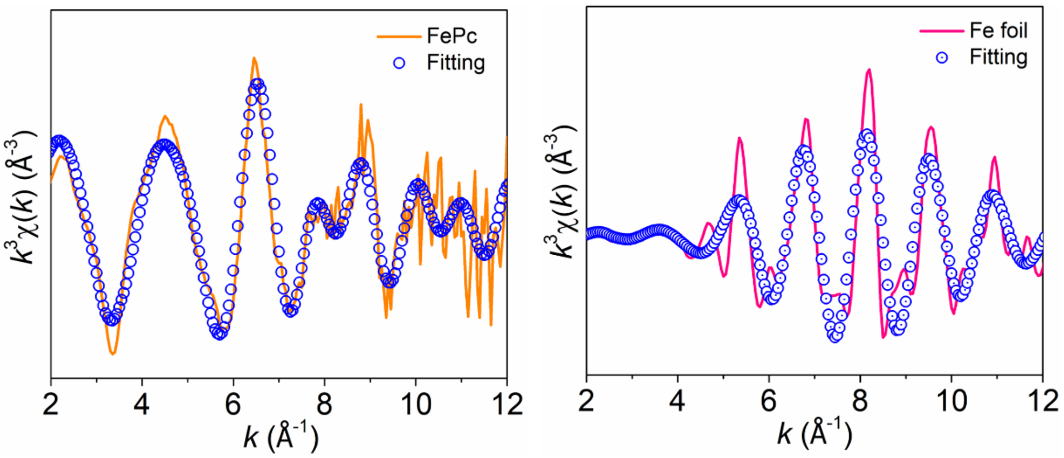

Figure S19. Fe K-edge EXAFS spectra in *k* space and the corresponding fitting curves for the FePc and Fe foil reference samples.

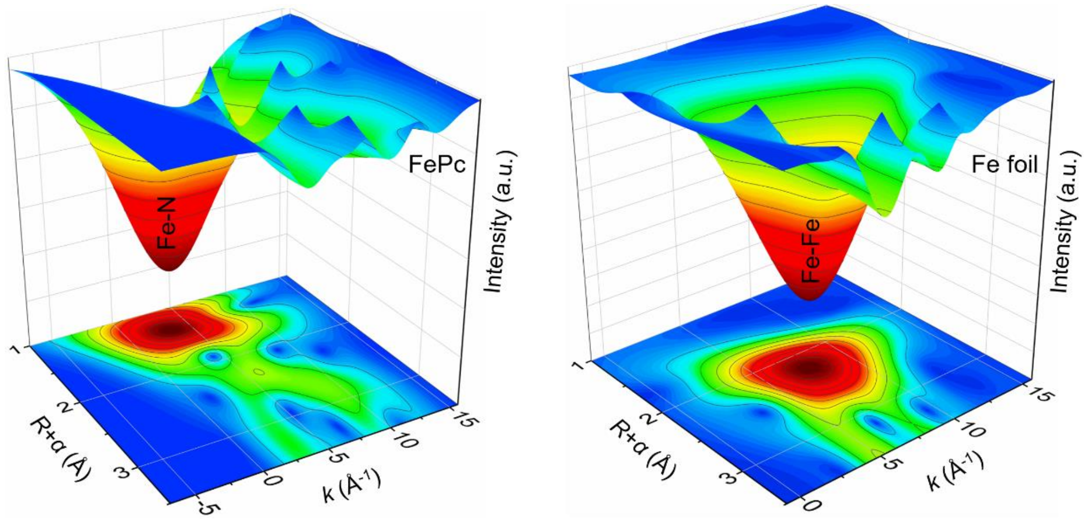

Figure S20. k^3-weighted Fe K-edge WT-EXAFS contour plots for the FePc and Fe foil reference samples.

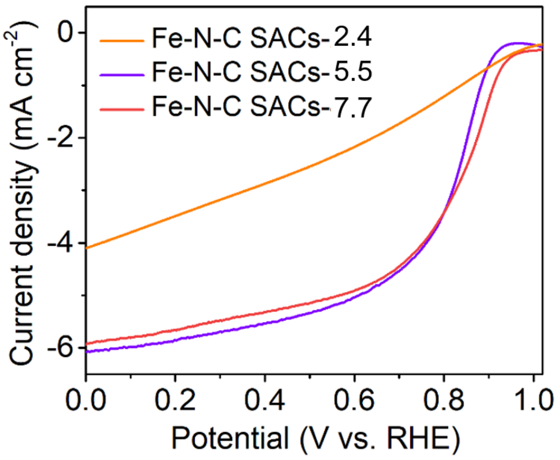

Figure S21. ORR LSV curves in O_2-saturated 0.1 M KOH for Fe-N-C/Fe_3C-2.4, Fe-N-C/Fe_3C-5.5 and Fe-N-C/Fe_3C-7.7.

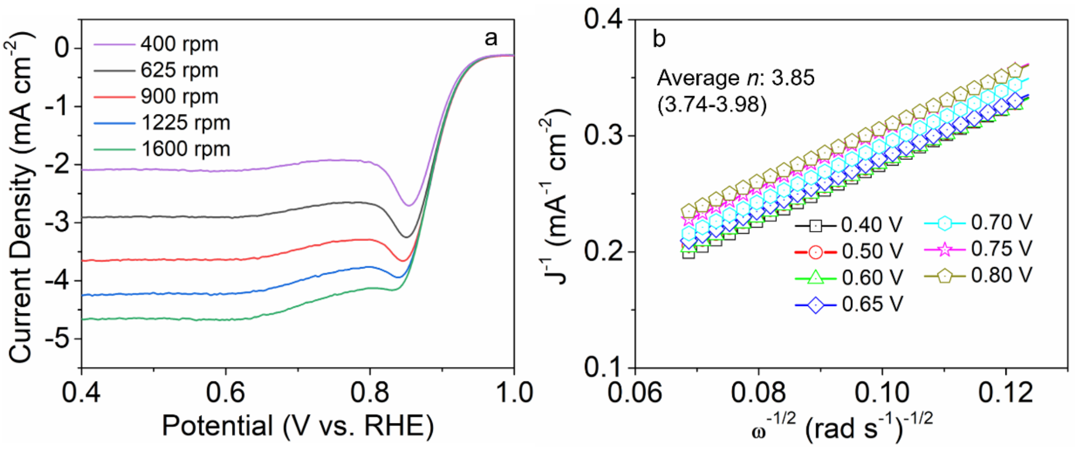

Figure S22. (a) The LSV polarization curves of Fe-N-C/Fe_3C-op at different rotating speeds and (b) corresponding *K*-*L* plots.

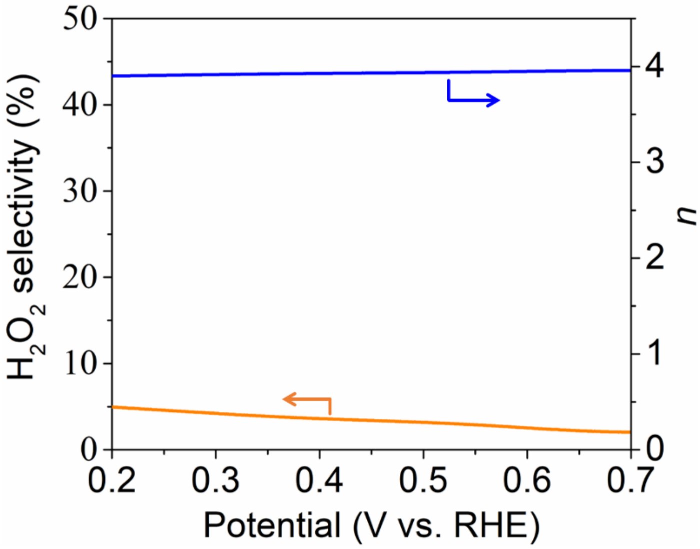

Figure S23. Calculated *n* and H_2O_2 selectivity for ORR on the Fe-N-C/Fe_3C-op electrocatalyst between 0.2 and 0.7 V.

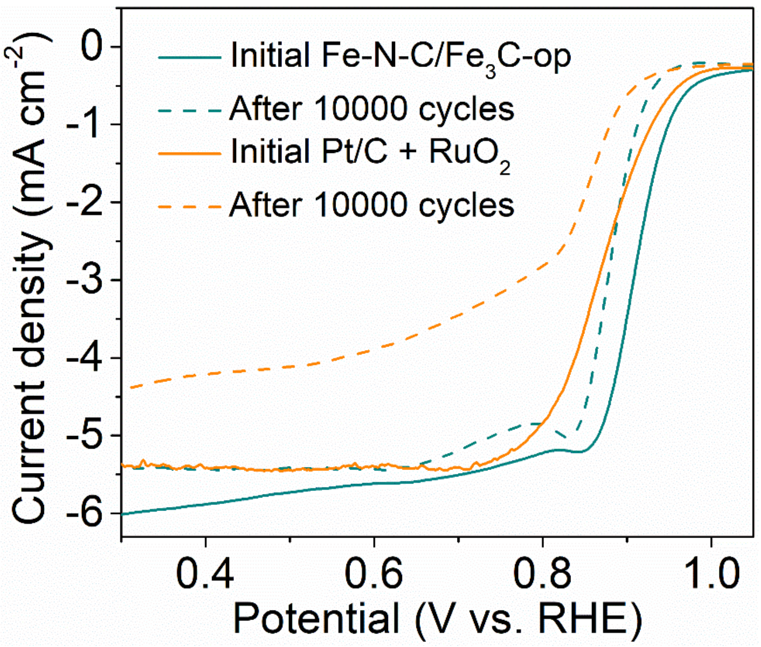

Figure S24. Steady-state ORR polarization curves of for the Fe-N-C/Fe_3C-op and Pt/C + RuO_2 electrocatalysts before and after 10000 potential scanning cycles at 50 mV s^{-1}.

As can be clearly seen, the polarization curves for Fe-N-C/Fe_3C-op recorded after 10000 cycles showed less degradation in $E_{1/2}$ and the limiting current density compared to the Pt/C + RuO_2 electrocatalyst, suggesting its superior durability in the alkaline electrolyte.

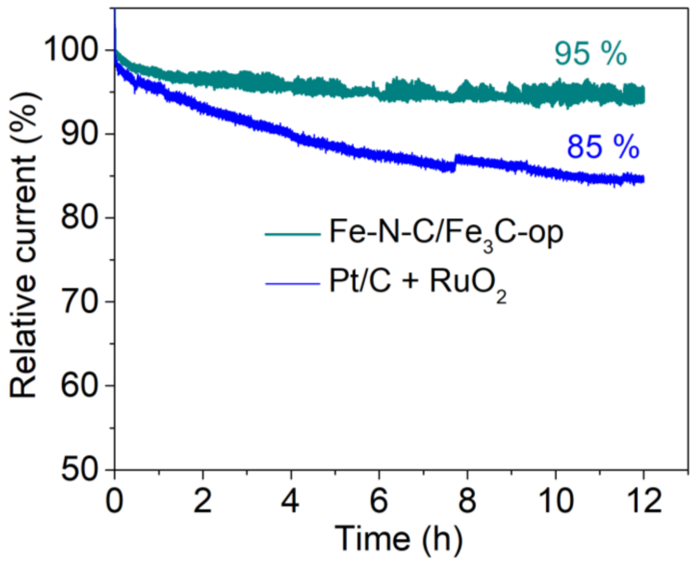

Figure S25. The *i-t* test for Fe-N-C/Fe_3C-op and Pt/C + RuO_2 electrocatalyst in O_2-saturated 0.1 M KOH electrolyte.

The results indicate that the performance of the Fe-N-C/Fe_3C-op electrocatalyst decrease by only 5 % after the 12 h test, whereas a significantly higher 15 % current loss was observed for Pt/C + RuO_2 electrocatalyst, indicating the superior stability of Fe-N-C/Fe_3C-op.

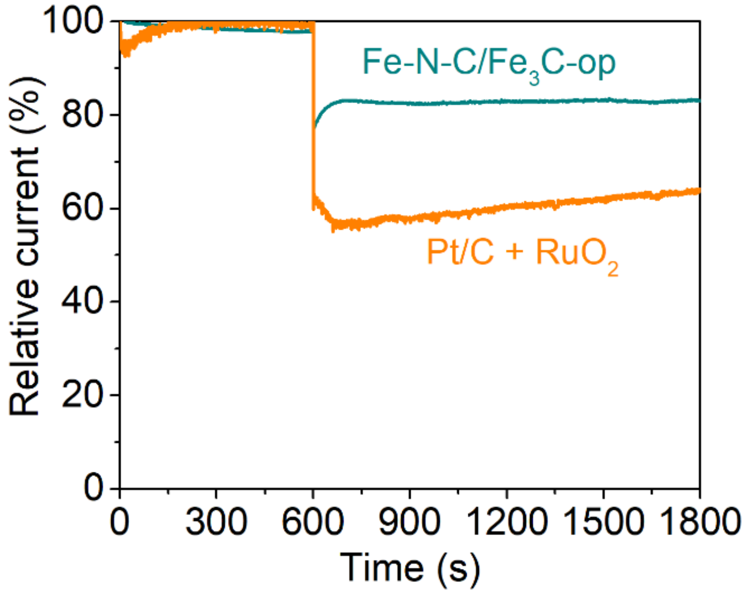

Figure S26. Chronoamperometric test assessing the methanol tolerance ability of Fe-N-C/Fe_3C-op and the commercial Pt/C + RuO_2 electrocatalyst. with and without the addition of 1.0 M CH_3OH, in the O_2-saturated 0.1 M KOH electrolyte.

The chronoamperometric data shows that the ORR activity of Fe-N-C/Fe_3C-op did not significantly decrease after the addition of methanol. In stark contrast, the electrocatalytic performance of the Pt/C + RuO_2 benchmark electrocatalyst decreased significantly following methanol addition, implying Fe-N-C/Fe_3C-op possessed better anti-poisoning ability than Pt/C + RuO_2.

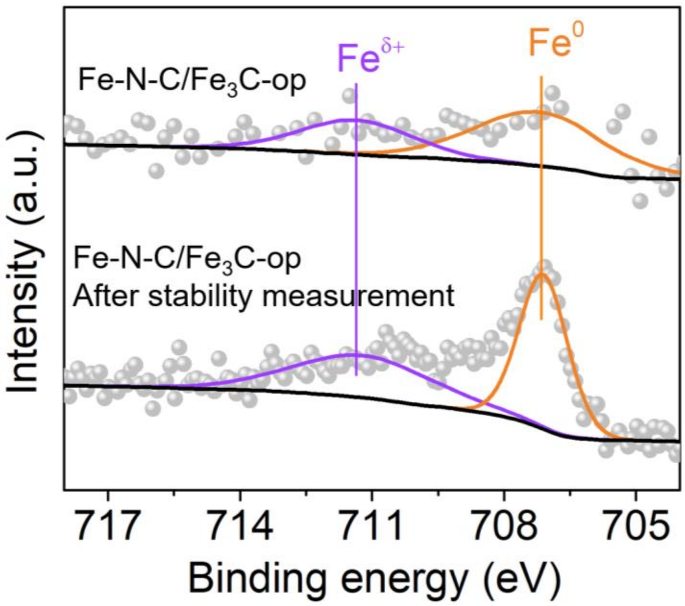

Figure S27. Deconvolution of the high-resolution Fe 2p$_{3/2}$ XPS spectra for Fe-N-C/Fe$_3$C-op after stability measurements.

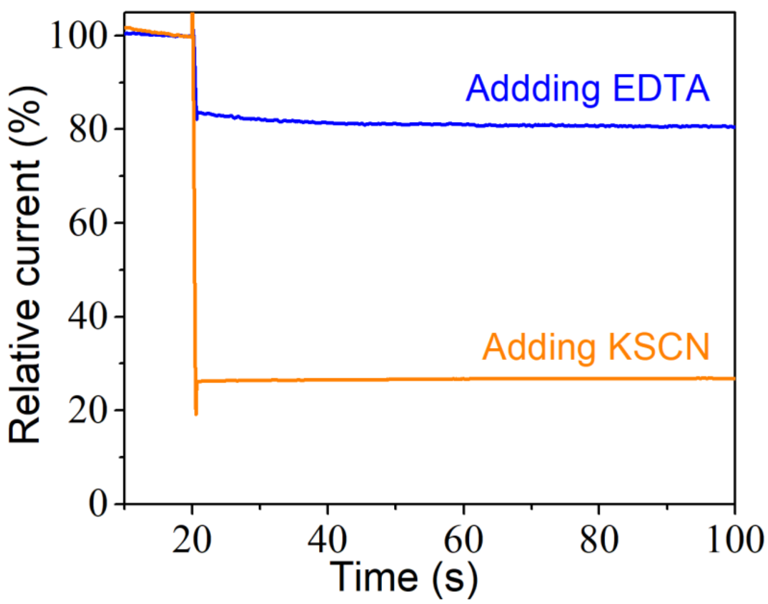

Figure S28. Demonstration of the synergistic effect between Fe-N_4-C and Fe_3C sites for Fe-N-C/Fe_3C-op electrocatalyst before and after the addition of thiocyanate ions (SCN^-) and EDTA ions in 0.1 M KOH electrolyte.

It is well-known that SCN^- can poison the active site of both Fe single sites and Fe nanoparticles, while EDTA only selectively coordinate with Fe single site for poisoning.[3, 21-23] In this view, ORR experiments were conducted in a 0.1 M KOH electrolyte containing 10 mM SCN^- or 10 mM EDTA to probe active site for ORR in Fe-N-C/Fe_3C-op. On adding EDTA, the current decreased by ~20%, indicating that Fe single atom sites were involved in ORR. On adding SCN^-, a much larger current decrease was observed, demonstrating that both Fe-N_4-C site and Fe_3C species contribute to the excellent ORR catalytic performance of Fe-N-C/Fe_3C-op.

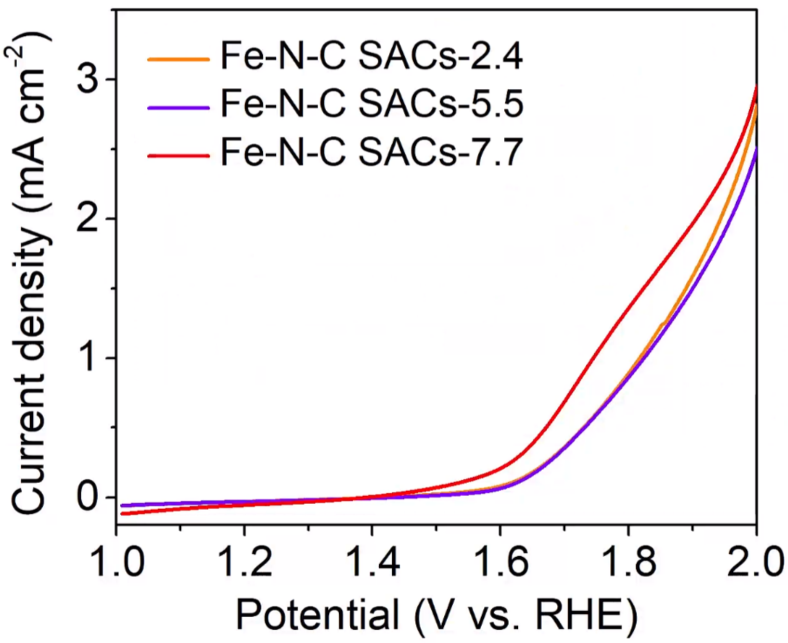

Figure S29. OER LSV curves the Fe-N-C SACs-x electrocatalysts.

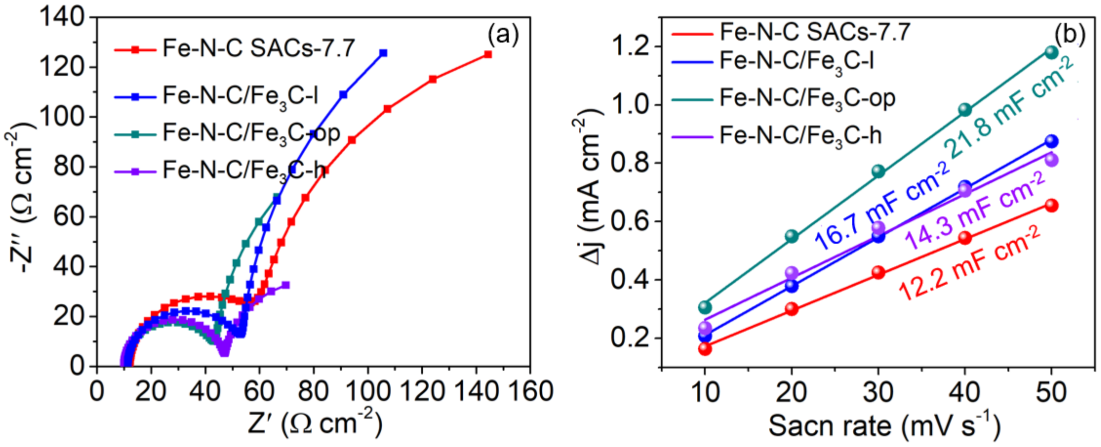

Figure S30. (a) Nyquist plots and (b) C_{dl} plots for these obtained electrocatalyst.

To further explore the potential reason for the improved performance, electrochemical impedance spectroscopy measurements (EIS) were conducted to study the charge transfer process. As can be seen in Figure S30a, Fe-N-C/Fe_3C-op exhibits the smallest charge transfer resistance in comparison with other samples. Also, cyclic voltammetry in the non-Faraday region was conducted to calculate the double layer capacitance (C_{dl}), which was positively correlated with the electrochemical surface area (ECSA). The higher value for Fe-N-C/Fe_3C-op indicates its larger electrochemical surface area and more active sites.

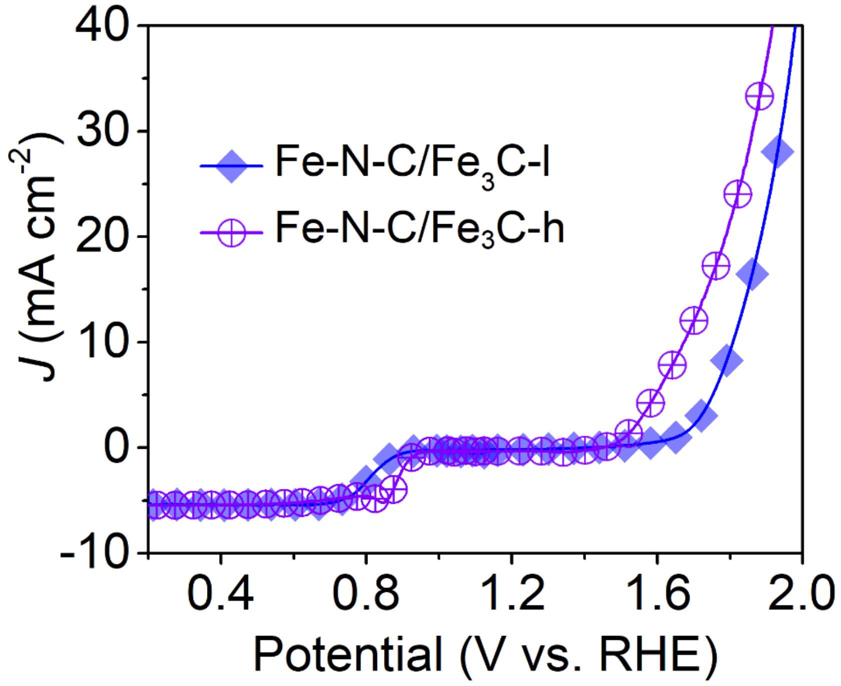

Figure S31. Bifunctional LSV curves of Fe-N-C/Fe_3C-l and Fe-N-C/Fe_3C-h for ORR and OER.

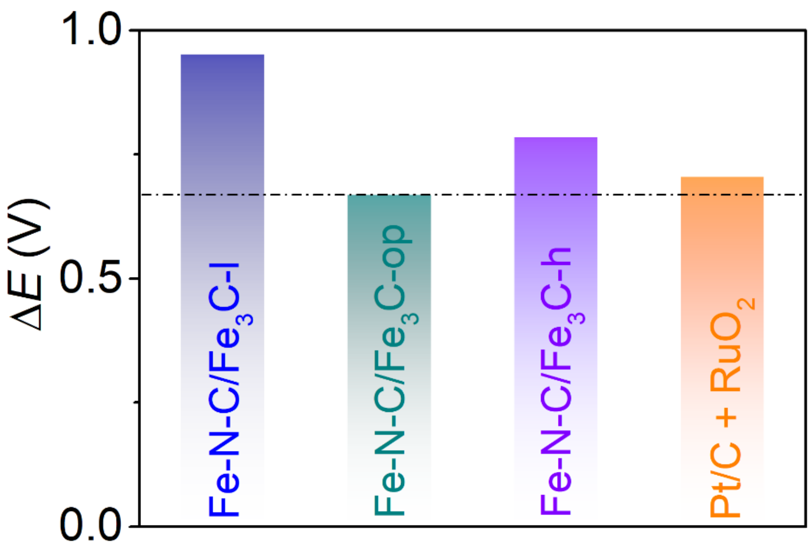

Figure S32. Diagram comparing the bifunctional electrocatalytic performance of Fe-N-C/Fe_3C-l, Fe-N-C/Fe_3C-op, Fe-N-C/Fe_3C-h and Pt/C + RuO_2 electrocatalysts in terms of ΔE.

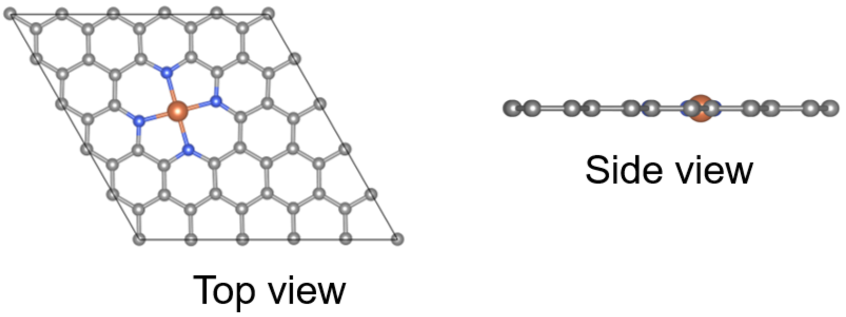

Figure S33. Model structure of $Fe-N_4-C$ constructed on graphene for theoretical calculation in this work.

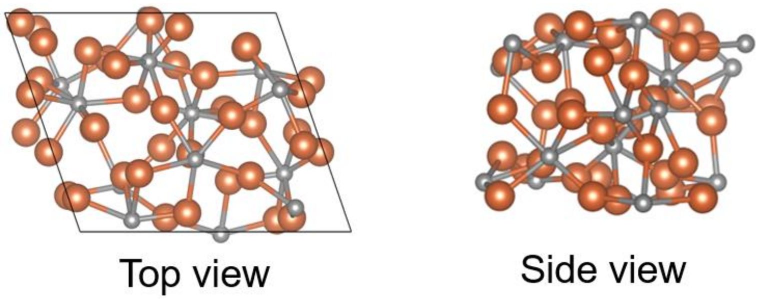

Figure S34. Structure of Fe_3C with (211) crystal face constructed on graphene for theoretical calculation in this work.

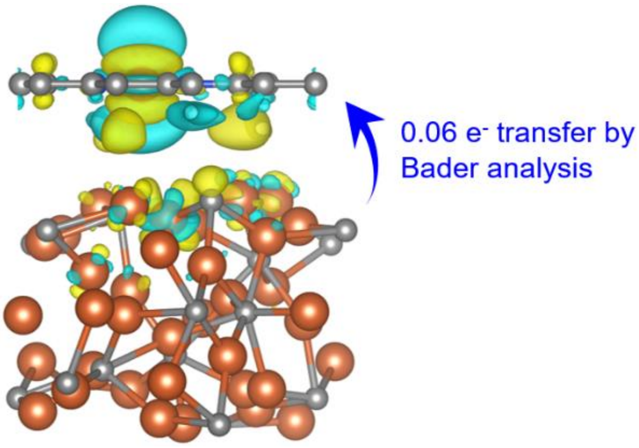

Figure S35. Charge density distributions and Bader charge transfer for Fe-N-C/Fe_3C model.

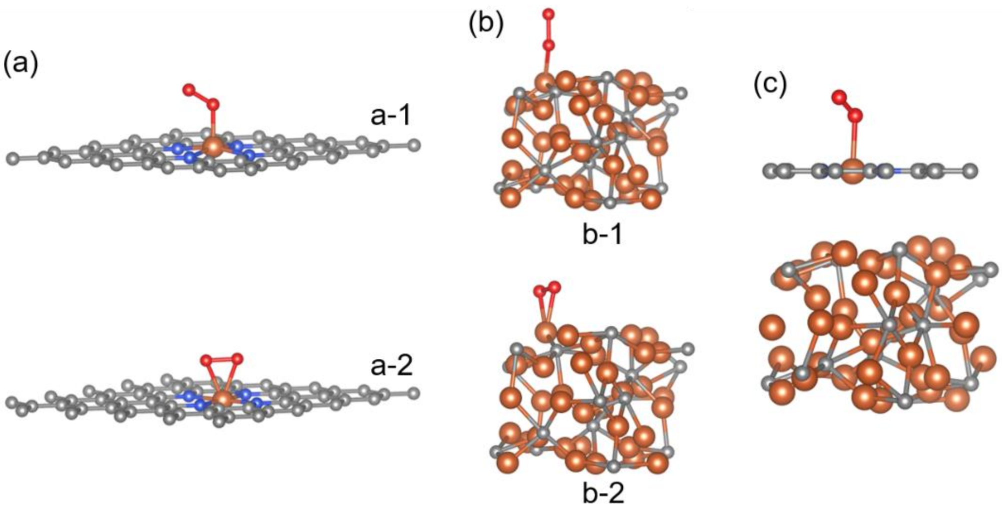

Figure S36. Optimized O_2 adsorption configurations in (a) Fe-N_4-C, (b) Fe_3C, and (c) Fe-N_4-C/Fe_3C. Note that a-1 and b-1 are the end-on configurations, whereas a-2 and b-2 are the side-on configurations.

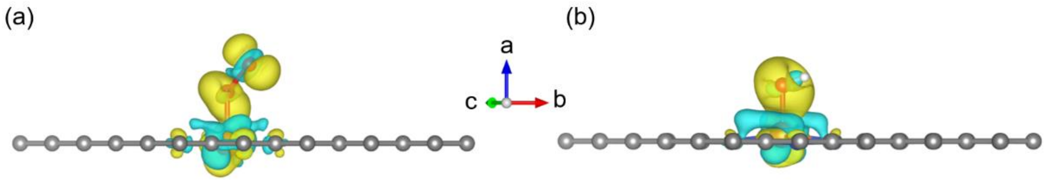

Figure S37. (a) Electron density difference plots for the Fe-N_4-C model with O_2^* and (b) OH^*, respectively. Cyan and yellow contours denotes charge depletion and accumulation, respectively, in real space.

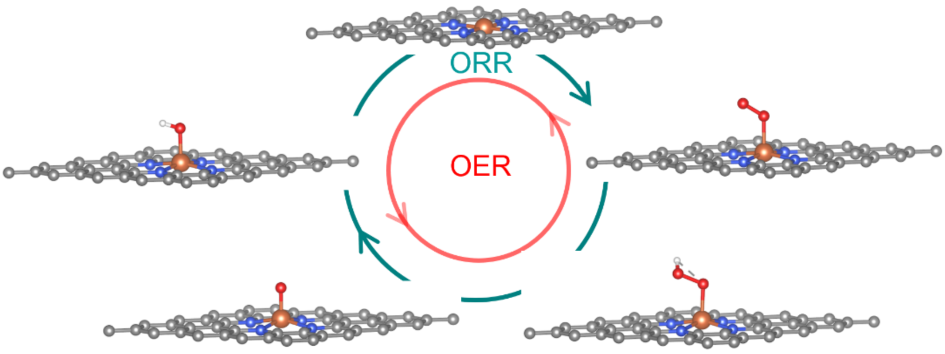

Figure S38. Optimized structures of the ORR and OER intermediates on Fe-N_4-C.

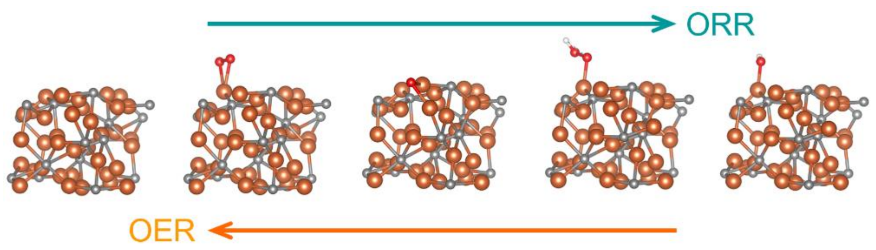

Figure S39. Optimized structures of the ORR and OER intermediates on Fe_3C.

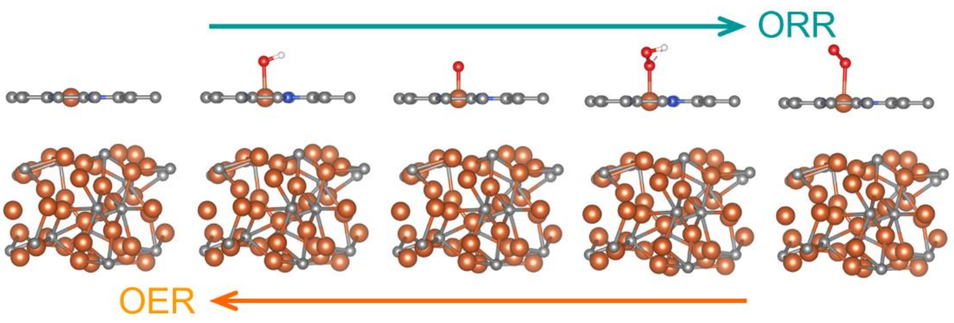

Figure S40. Optimized structures of the ORR and OER intermediates on $Fe\text{-}N_4\text{-}C/Fe_3C$.

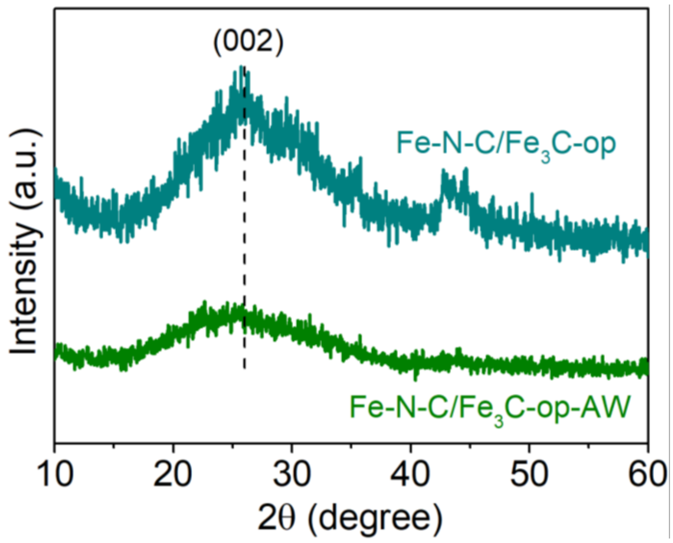

Figure S41. XRD patterns for Fe-N-C/Fe_3C-op and Fe-N-C/Fe_3C-op-AT. After acid washing, only the (002) graphite peak can be observed for Fe-N-C/Fe_3C-op-AT.

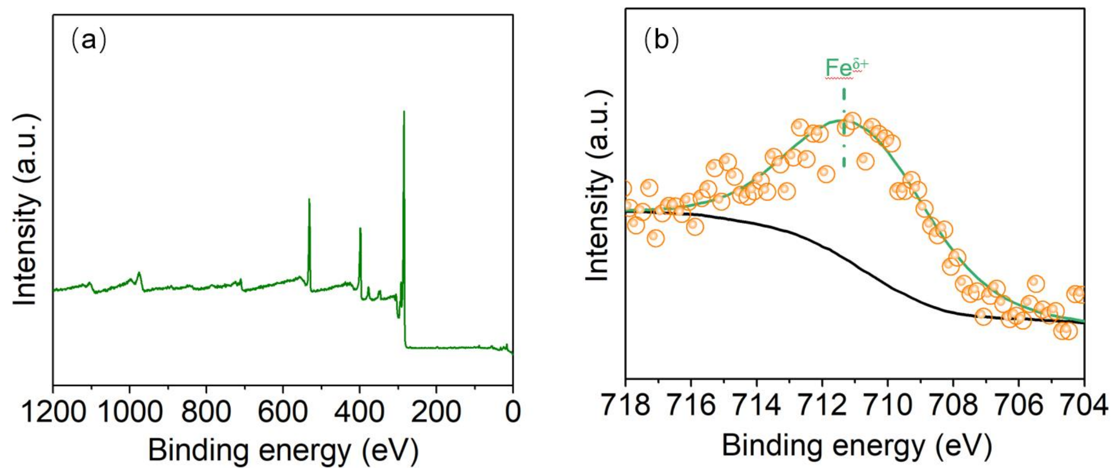

Figure S42. (a) XPS survey spectrum and (b) fitted Fe $2p_{3/2}$ spectrum for Fe-N-C/Fe_3C-op-AT.

For the Fe-N-C/Fe_3C-op-AT catalyst, only $Fe^{\delta+}$ was detected, indicating the removal of Fe_3C species.

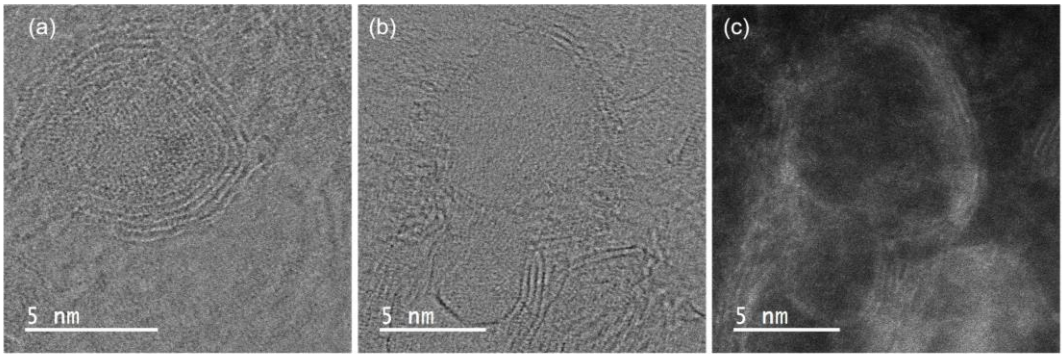

Figure S43. (a) and (b) Annular bright-field and (c) dark-field STEM images of acid treated Fe-N-C/Fe_3C-op-AT.

After the H_2SO_4 treatment, no metallic Fe_3C species were observed with only hollow onion-like structures consisting of few-layer graphitic carbon remaining. These results are consistent with the findings in Figure S38 and S39.

Table S1. A summary of the Fe loading for Fe-N-C SACs-x, Fe-N-C/Fe_3C-l, Fe-N-C/Fe_3C-op, Fe-N-C/Fe_3C-h and Fe-N-C/Fe_3C-op after measurement, estimated by ICP-OES.

Samples	Average Fe content (wt%)
**Fe-N-C SACs-2.4**	2.4
**Fe-N-C SACs-5.5**	5.5
**Fe-N-C SACs-7.7**	7.7
**Fe-N-C/Fe_3C-l**	11.2
**Fe-N-C/Fe_3C-op**	**14.9**
**Fe-N-C/Fe_3C-h**	23.1
**Fe-N-C/Fe_3C-op after measurement**	14.2

Table S2. EXAFS fitting parameters at the Fe K-edge for the as-prepared samples.

**Samples**	**Bond type**	***CN*** [a]	***R*** **(Å)** [b]	**σ^2 (10^{-3}Å^2)** [c]	**ΔE_o (eV)** [d]	**R factor** [e]
**Fe foil**	Fe-Fe	8	2.47±0.01	0.0040±0.0007	6.3±1.2	0.0060
**FePc**	Fe-N	4.0±0.1	2.01±0.03	0.0118±0.0035	8.4±1.6	0.0076
	Fe-N-C	8.0±0.3	2.95±0.02	0.0116±0.0052		
**Fe-N-C/Fe_3C-op**	Fe-N	4.2±0.5	1.94±0.01	0.0131±0.0016	0.4±1.1	0.0048

[a]*CN*: the coordination number of Fe atom; [b]*R*: the average bond length; [c]σ^2: Debye-Waller factors; [d]ΔE_0: the inner potential correction; [e]*R* factor: the evaluation index for the goodness of fittings.

Table S3. The content of different Fe species in Fe-N-C/Fe_3C-l, Fe-N-C/Fe_3C-op and Fe-N-C/Fe_3C-h samples.

Samples	Fe-N-C area (%)	Fe_3C area (%)	Ratio of Fe_3C to Fe-N-C content
**Fe-N-C/Fe_3C-l**	62.6	37.4	0.60
**Fe-N-C/Fe_3C-op**	48.7	51.3	1.05
**Fe-N-C/Fe_3C-h**	19.2	80.8	2.42

Table S4. ORR and OER performance for the Fe-N-C SACs-x, Fe-N-C/Fe_3C-l, Fe-N-C/Fe_3C-op, Fe-N-C/Fe_3C-h, Pt/C + RuO_2 electrocatalysts in this work tested in 0.1 M KOH electrolyte.

Samples	$E_{1/2}$ (V)	E_{j10} (V)	ΔE (V)
Fe-N-C SACs-2.4	-	-	-
Fe-N-C SACs-5.5	0.827	-	-
Fe-N-C SACs-7.7	0.837	-	-
Fe-N-C/Fe_3C-l	0.857	1.809	0.952
Fe-N-C/Fe_3C-op	0.911	1.579	0.668
Fe-N-C/Fe_3C-h	0.889	1.673	0.784
Pt/C + RuO_2	0.874	1.630	0.756

Table S5. Comparison of the bifunctional ORR/OER activity and corresponding ZABs performance of Fe-N-C/Fe_3C-op and other recently reported bifunctional ORR/OER catalysts activity in an O_2-saturated 0.10 M KOH electrolyte.

Catalyst	$E_{1/2}$ (V)	E_{j10} (V)	ΔE (V)	Powder density (mW cm^{-2})	Specific capacity (mAh g_{Zn}^{-1})	ref.
Fe-N-C/Fe_3C-op	0.911	1.579	0.668	137.4	818.1	this work
Pt/C + RuO_2	0.874	1.630	0.756	123.7	710.4	
Co_3O_4-NrmGO	0.83	1.54	0.71	-	-	*Nat. Mater*. **10**, 780-786 (2011)
Co_3O_4/rmGO	0.79	1.62	0.83	-	-	*Nat. Mater.* **10**, 780 (2011)
CNT/graphene hybrid	0.76	1.51	0.75	-	-	*Nat. Nanotech*. **7**, 394 (2012)
NPMC	0.85	~2.0	1.15	-	-	*Nat. Nanotech*. **10**, 444-452 (2015)
SC CoO	0.85	1.56	0.71	-	-	*Nat. Commun*. **7**, 12876 (2016)
NCNTFs	0.87	1.6	0.73	-	-	*Nat. Energy*. **1**, 15006 (2016)
Ni-NHGF	0.86	1.56	0.70	-	-	*Nat. Catal*. **1**, 6372 (2018)
$Nd_{1.5}Ba_{1.5}CoFeM_nO_{9-\delta}$	0.89	1.59	0.70	-	-	*Sci. Adv*. **4**, eaap9360 (2018)
PdMo bimetallene	0.95	1.70	0.75			*Nature* **574**, 81-85 (2019)
Fe,Mn/N-C	0.928	1.62	0.692	160.8	902	*Nat. Commun*. **12**, 1734 (2021)
MoS_2@Fe-N-C NSs	0.84	1.70	0.86	78	442	*Proc. Natl. Acad. Sci. U.S.A.* **118**, e2110036118 (2021)
CoNC SAC	0.86	1.65	0.79	161.8	795	*Sci. Adv.* **8**, eabn5091 (2022)
(Co, Fe)$_3$N	0.81	1.62	0.81	234	-	*Nat.Commun*. **11**, 1952 (2020)
Co_4N/CNW/CC	0.8	1.54	0.74	174	774	*J. Am. Chem. Soc*. **138**, 10226 (2016)
NC@Co-NGC DSNCs	0.82	1.63	0.82	-	-	*Adv. Mater*. **29**, 1700874 (2017)
Enzyme-inspired Fe porphyrin/CNT	0.84	1.73	0.89	132.9	785.9	*Angew. Chem., Int. Ed.* **60**, 7576 (2021)
GNCNTs-4	0.85	1.60	0.75	253	728	*Adv. Funct. Mater*. **30**, 1906081 (2020)
Co@hNCTs-800	0.87	1.63	0.76	149	746	*Nano Energy* **71**, 2211

						(2020)
FeMn-DSAC	0.922	1.635	0.713	184	737	*Angew. Chem. Int. Ed.* **61**, e202115219 (2022)
ZOMC	0.85	2.56	0.71	221	772	*Adv. Mater*. **32**, 2002170 (2020)
OLC/Co-N-C	0.855	1.58	0.725	238	790	*Angew. Chem., Int. Ed.* **60**, 12759 (2021)
Co tetra (imidazolyl)porphyrin @ZIF-67	0.79	1.646	0.856	220	654	*Angew. Chem., Int. Ed.* **60**, 8472 (2021)
MnO@CNT@Co-N/C	0.81	1.62	0.81	200.8	802.7	*J. Mater. Chem. A* **9**, 22533 (2021)
MnO/Co/PGC	0.78	1.537	0.757	172	-	*Adv. Mater*. **31**, 1902339 (2019)
FeNiPNCH	0.75	1.48	0.73	250	-	*J. Am. Chem. Soc*. **141**, 7906-7916 (2019)
N-CoS_2 YSSs	0.81	1.508	0.698	81	744	Adv. Sci. 2020, 7, 2001178
CNT@SAC-Co/NCP	0.87	1.61	0.74	172	-	Adv. Funct. Mater. 2021, 31, 2103360
0.4-Ru@NG750	0.826	1.602	0.776	-	-	*ACS Catal*. **9**, 9897-9904 (2019)
PtPdCo	0.96	1.65	0.69	-	-	*Adv. Mater*. **30**, 1802136 (2018)
RuO_2-Mn_2O_3	0.78	1.67	0.89	-	-	*Nano Lett*. **17**, 2076-2083 (2016)
N-GRW	0.84	1.59	0.75	-	-	*Sci. Adv*. **2**, eaap1501122 (2016)
Meso/micro-FeCoNx-CN-30	0.886	1.670	0.784	150	-	*Angew. Chem., Int. Ed.* **57**, 1856 (2018)
CoNi-SAs/NC	0.760	1.570	0.810	101.4	750.9	*Adv. Mater*. **31**, 1905622 (2019)
C_{60}-SWCNT	0.84	1.69	0.85	-	-	*J. Am. Chem. Soc*. **141**, 1165811666 (2019)
NiN_4/GHSs/FeN_4	0.830	1.620	0.790	-	777.6	*Adv. Mater*. **32**, 2003134 (2020)
S,N-Fe/N/CCNT	0.850	1.600	0.750	102.7	-	*Angew. Chem. Int. Ed.* **56**, 610–614 (2017)
LT-$LiCoO_2$	0.65	1.6	0.95	-	-	*Nat.Commun*. **5**, 3949 (2014)
Pt-$CoSe_{2-x}$	0.83	1.56	0.73	-	-	*Adv. Mater*. **31**, 1805581 (2019)
Co_2Fe_2@ NC	0.85	1.65	0.80	-	812.5	*J. Am.Chem. Soc*. **142**, 7116-7127 (2020)

Fe_2-Co_1GNCL	0.846	1.58	0.734	-	-	*Angew. Chem. Int. Ed.* **59**, 2-11 (2020)
$Pb_2Ru_2O_{6.5}$	0.81	1.65	0.84	-	-	*Energy Environ. Sci.*, 2017, 10, 129-136
CPG-900	0.65	2.0	1.35	-	-	*Nat. Commun.* **5**, 5285 (2014)
$Cu_{6.81}$-CoFS	0.80	1.54	0.74	255	-	*Energy Environ. Sci.* **14**, 5035-5043 (2021)
Fe-N/C-800	0.80	1.65	0.85	-	-	*J. Am. Chem. Soc.* **136**, 1102 (2014)
$MnCo_2O_4$	0.85	1.68	0.83	-	-	*Angew. Chem. Int. Ed.* **56**, 14977-14981 (2017)
S-CFZ	0.85	1.53	0.68	-	-	*J. Am. Chem. Soc.* **144**, 4783-4791 (2022)
ZIF8-Te-1000	0.83	1.60	0.77	-	-	*J. Am. Chem.Soc.* **136**, 14385 (2014)
sulphur-doped graphene	0.66	1.35	0.69	-	-	*Angew. Chem. Int. Ed.* **54**, 1888 (2015)
Mesoporous N-doped carbon	0.85	1.68	0.83	-	-	*Nat. Commun.* **5**, 5974 (2014)
RuO_x-nc@-Co_3O_4-250	0.8	1.51	0.71	150		*Energy Storage Materials* **32**, 20-29 (2020).
graphene-MOF composite	-	-	0.74	-	-	*J. Am. Chem.Soc.* **134**, 6707 (2012)
Fe,Co,N-C	0.9	1.64	0.74	158	-	*ACS Nano* **16**, 7890-7903 (2022).
ZIF-derived porous carbons	-	-	0.68	-	-	*J. Am. Chem.Soc.* **136**, 6790 (2014)
P-doped ZIF8-derived carbons	0.71	1.48	0.77	-	-	*J. Am. Chem.Soc.* **136**, 14385 (2014)
P-doped ordered mesoporous carbon	0.82	1.55	0.73	~130		*Angew. Chem. Int. Ed.* **54**, 9230 (2015)
Ni–MnO/rGO	0.78	1.60	0.82	123	758	*Adv. Mater.* **30**, 1704609 (2018).
N/Co-doped PCP//NRGO	0.86	1.68	0.82	-	-	*Adv. Funct. Mater.* **25**, 872 (2015)
$Co_3FeS_{1.5}(OH)_6$	0.721	1.588	0.867	113.1	898	*Adv. Mater.* **29**, 1702327 (2017).

Table S6. Calculated O_2 adsorption energies on Fe-N_4-C, Fe_3C and Fe-N_4-C/Fe_3C models.

Model	O_2 adsorption energies (eV)	
	End-on	Side-on
Fe-N_4-C	-0.314	0.124
Fe_3C	-2.602	-2.985
Fe-N_4-C/Fe_3C	-0.708	/

The adsorption of O_2 on the catalyst surface is an essential step for the ORR reaction. Thus, O_2 adsorption energies on the three catalyst models were investigated, considering both side-on and end-on configurations. The calculations show that O_2 adsorption on Fe_3C surface will likely occur via a side-on configuration, whereas end-on configuration is more favorable for both the Fe-N_4-C and Fe-N_4-C/Fe_3C models. Further, after the introduction of Fe_3C, O_2 adsorption on the Fe-N_4-C surface was promoted (-0.708 eV for Fe-N_4-C/Fe_3C vs. -0.314 eV for Fe-N_4-C). Although the Fe_3C model offered the highest adsorption energies among the three models studied due to its electron-rich characteristics, the very strong O_2 adsorption ability was likely not beneficial for ORR or OER according to the Sabatier principle[24, 25].

Table S7. Calculated O-O bond lengths for adsorbed O_2 on Fe-N_4-C, Fe_3C and Fe-N_4-C/Fe_3C models.

Model	O-O (Å)	
	End-on	Side-on
Fe-N_4-C	1.265	1.359
Fe_3C	1.321	1.374
Fe-N_4-C/Fe_3C	1.285	/
O_2 molecule	1.234	

The O-O bond length for O_2 adsorbed on Fe-N_4-C/Fe_3C was elongated in comparison with that of Fe-N_4-C model, signifying that O_2 molecules were more effectively activated on the Fe-N_4-C/Fe_3C surface. Consistent to the results in Table S6, O_2 adsorption on Fe_3C was the strongest, resulting in the largest O-O lengths for both end-on and side-on configurations.

Table S8. Calculated ICOHP values for $Fe\text{-}N_4\text{-}C$ and $Fe\text{-}N_4\text{-}C/Fe_3C$ models.

Model	ICOHP (O_2^*)	ICOHP (OH^*)
$Fe\text{-}N_4\text{-}C$	−0.75	−2.96
$Fe\text{-}N_4\text{-}C/Fe_3C$	−1.79	−2.34

References

[1] H. Song, M. Wu, Z. Tang, J.S. Tse, B. Yang, S. Lu, *Angew. Chem. Int. Ed.* **2021**, *60*, 7234-7244.

[2] W. Li, Y. Liu, M. Wu, X. Feng, S.A.T. Redfern, Y. Shang, X. Yong, T. Feng, K. Wu, Z. Liu, B. Li, Z. Chen, J.S. Tse, S. Lu, B. Yang, *Adv. Mater.* **2018**, *30*, 1800676.

[3] L. Zhao, Y. Zhang, L.-B. Huang, X.-Z. Liu, Q.-H. Zhang, C. He, Z.-Y. Wu, L.-J. Zhang, J. Wu, W. Yang, L. Gu, J.-S. Hu, L.-J. Wan, *Nat. Commun.* **2019**, *10*, 1278.

[4] P. Hohenberg, W. Kohn, *Physical Review* **1964**, *136*, B864-B871.

[5] W. Kohn, L.J. Sham, *Physical Review* **1965**, *140*, A1133-A1138.

[6] G. Kresse, J. Furthmüller, *Phys. Rev. B* **1996**, *54*, 11169-11186.

[7] G. Kresse, D. Joubert, *Phys. Rev. B* **1999**, *59*, 1758-1775.

[8] Y. Zhang, W. Yang, *Phys. Rev. Lett.* **1998**, *80*, 890-890.

[9] J.P. Perdew, J.A. Chevary, S.H. Vosko, K.A. Jackson, M.R. Pederson, D.J. Singh, C. Fiolhais, *Phys. Rev. B* **1992**, *46*, 6671-6687.

[10] H.J. Monkhorst, J.D. Pack, *Phys. Rev. B*, **1976**, *13*, 5188-5192.

[11] H.W. Kim, M.B. Ross, N. Kornienko, L. Zhang, J. Guo, P. Yang, B.D. McCloskey, *Nat. Catal.* **2018**, *1*, 282-290.

[12] S. Lu, Y. Shi, W. Zhou, Z. Zhang, F. Wu, B. Zhang, *J. Am. Chem. Soc.* **2022**, *144*, 3250-3258.

[13] C. Xia, Y. Qiu, Y. Xia, P. Zhu, G. King, X. Zhang, Z. Wu, J.Y. Kim, D.A. Cullen, D. Zheng, P. Li, M. Shakouri, E. Heredia, P. Cui, H.N. Alshareef, Y. Hu, H. Wang, *Nat. Chem.* **2021**, *13*, 887-894.

[14] Y. Zheng, L. Lin, B. Wang, X. Wang, *Angew. Chem. Int. Ed.* **2015**, *54*, 12868-12884.

[15] J. Li, M.T. Sougrati, A. Zitolo, J.M. Ablett, I.C. Oğuz, T. Mineva, I. Matanovic, P. Atanassov, Y. Huang, I. Zenyuk, A. Di Cicco, K. Kumar, L. Dubau, F. Maillard, G. Dražić, F. Jaouen, *Nat. Catal.* **2021**, *4*, 10-19.

[16] S. Liu, C. Li, M.J. Zachman, Y. Zeng, H. Yu, B. Li, M. Wang, J. Braaten, J. Liu, H.M. Meyer, M. Lucero, A.J. Kropf, E.E. Alp, Q. Gong, Q. Shi, Z. Feng, H. Xu, G. Wang, D.J. Myers, J. Xie, D.A. Cullen, S. Litster, G. Wu, *Nat. Energy* **2022**, *7*, 652-663.

[17] F. Kong, X. Cui, Y. Huang, H. Yao, Y. Chen, H. Tian, G. Meng, C. Chen, Z. Chang, J. Shi, *Angew. Chem. Int. Ed.* **2022**, *61*, e202116290.

[18] X. Hai, S. Xi, S. Mitchell, K. Harrath, H. Xu, D.F. Akl, D. Kong, J. Li, Z. Li, T. Sun, H. Yang, Y. Cui, C. Su, X. Zhao, J. Li, J. Pérez-Ramírez, J. Lu, *Nat. Nanotech.* **2022**, *17*, 174-181.

[19] J. Chang, X. Song, C. Yu, H. Huang, J. Hong, Y. Ding, H. Huang, J. Yu, X. Tan, Z. Zhao, J. Qiu, *Nano Energy*, **2020**, *69*, 104377.

[20] J. Chang, X. Song, C. Yu, J. Yu, Y. Ding, C. Yao, Z. Zhao, J. Qiu, *Adv. Funct. Mater.* **2020**, *30*, 2006270.

[21] X. Wan, Q. Liu, J. Liu, S. Liu, X. Liu, L. Zheng, J. Shang, R. Yu, J. Shui, *Nat. Commun.* **2022**, *13*, 2963.

[22] C. Tang, L. Chen, H. Li, L. Li, Y. Jiao, Y. Zheng, H. Xu, K. Davey, S.-Z. Qiao, *J. Am. Chem. Soc.*, **2021**, *143*, 7819-7827.

[23] W. Wan, Y. Zhao, S. Wei, C.A. Triana, J. Li, A. Arcifa, C.S. Allen, R. Cao, G.R. Patzke, *Nat. Commun.* **2021**, *12*, 5589.
[24] S. Hu, W.-X. Li, *Science* **2021**, *374*, 1360-1365.
[25] D.-Y. Kuo, H. Paik, J. Kloppenburg, B. Faeth, K.M. Shen, D.G. Schlom, G. Hautier, J. Suntivich, *J. Am. Chem. Soc.* **2018**, *140*, 17597-17605.
